# Classifying RNA-Binding Proteins Based on Electrostatic Properties

**DOI:** 10.1371/journal.pcbi.1000146

**Published:** 2008-08-08

**Authors:** Shula Shazman, Yael Mandel-Gutfreund

**Affiliations:** Faculty of Biology, Technion—Israel Institute of Technology, Haifa, Israel; Duke University, United States of America

## Abstract

Protein structure can provide new insight into the biological function of a protein and can enable the design of better experiments to learn its biological roles. Moreover, deciphering the interactions of a protein with other molecules can contribute to the understanding of the protein's function within cellular processes. In this study, we apply a machine learning approach for classifying RNA-binding proteins based on their three-dimensional structures. The method is based on characterizing unique properties of electrostatic patches on the protein surface. Using an ensemble of general protein features and specific properties extracted from the electrostatic patches, we have trained a support vector machine (SVM) to distinguish RNA-binding proteins from other positively charged proteins that do not bind nucleic acids. Specifically, the method was applied on proteins possessing the RNA recognition motif (RRM) and successfully classified RNA-binding proteins from RRM domains involved in protein–protein interactions. Overall the method achieves 88% accuracy in classifying RNA-binding proteins, yet it cannot distinguish RNA from DNA binding proteins. Nevertheless, by applying a multiclass SVM approach we were able to classify the RNA-binding proteins based on their RNA targets, specifically, whether they bind a ribosomal RNA (rRNA), a transfer RNA (tRNA), or messenger RNA (mRNA). Finally, we present here an innovative approach that does not rely on sequence or structural homology and could be applied to identify novel RNA-binding proteins with unique folds and/or binding motifs.

## Author Summary

Gene expression in all living organisms is regulated by a complex set of events at both transcriptional and posttranscriptional levels. RNA-binding proteins play a key role in posttranscriptional events including splicing, stability, transport, and translation. Nowadays, there is increasing evidence that many other cellular processes may be mediated by RNA. Identifying new proteins involved in interaction with RNA is thus essential to unraveling the cellular processes in which these interactions are involved. In the current study we present a successful computational approach for classifying RNA-binding proteins and distinguishing them from other proteins based on structural and electrostatic properties. We test the method on a unique protein domain, the RNA recognition motif (RRM), which mediates both RNA and protein interactions. We show that we can discriminate RNA-binding RRMs from protein-binding RRMs. Further, we demonstrate that we can classify known RNA-binding proteins based on their RNA target (mRNA, rRNA, or tRNA). Our method does not rely on any kind of evolutionary information and thus can be applied to identify RNA-binding proteins with novel modes of RNA recognition.

## Introduction

In recent years, there has been a growing appreciation for the importance of RNA and its interacting proteins. RNA-binding proteins (RBPs) function both in basic cellular processes and as key regulators of gene expression. Genome sequencing and analysis has identified many highly conserved noncoding RNAs [1] as well as numerous RBPs whose biological roles are still unknown. An increasing amount of new evidence on noncoding RNAs suggests that many other cellular processes may be mediated by RNA [2]. In most cases, RNA is found in complexes with proteins, either as large ribonucleoprotein complexes (such as the ribosome) or in more transient interactions (such as the helicase-RNA interactions) [3]. Identification of proteins involved in interaction with RNA is essential to unraveling the cellular processes in which these interactions are involved.

RBPs are characterized by a modular structure and are composed of multiple repeats that are built from a small number of basic domains that are arranged in various ways in order to satisfy their diverse functional requirements [4]. The RBPs can be classified into different families based on their basic binding motifs, for example: the RNA recognition motif (RRM), the KH domain, the double stranded RNA-binding domain (dsRBD), and the zinc finger motif [5]. Based on the first draft of the human genome, it was estimated that there are more than a thousand RBPs with known RNA-binding motifs in the genome. These numbers are expected to increase dramatically when considering all proteins that have RNA-binding capacities [6]. In recent years, new RRMs, such as the PAZ domain and the PIWI motif, which are found in the RNA-induced silencing complex (RISC), have been identified [7], revealing distinct, novel modes of RNA recognition [8]. An increasing amount of evidence on noncoding RNAs suggest that new RNA-binding motifs are yet to be discovered [9].

For many years, computational methods for identifying RNA-binding function based on structural information were not practical, due to the great diversity of the proteins and lack of structural information about them. With the exponential increase in the number of proteins being identified by genomics and proteomics projects, and specifically by structural genomics initiatives, predicting RNA-binding function from structure is now feasible. Since it is impractical to perform a functional assay for every uncharacterized protein, scientists have been turning to sophisticated computational methods for assistance in annotating the huge volume of sequence and structural data being produced. To date, many techniques are available for automatic function prediction, including: homology-based methods, phylogenetic methods, sequence patterns, structural similarity, structural patterns, methods based on genomic context, and microarray expression data [10]. Among these, several computational methods have concentrated specifically on predicting DNA-binding proteins from three-dimensional (3D) structures [11]–[16]. In addition, a couple of successful methods for prediction of RNA-binding function based on primary sequence were recently developed [17],[18].

The structural work of the last decade has elucidated the structures of many major RNA-binding protein families. Furthermore, the structures of proteins in complex with their RNA targets have shed light on how RNA recognition takes place [5]. Recently, several bioinformatics approaches have been applied for identifying RNA-binding sites on RBPs [19]–[22]. Here we present a machine learning approach to classifying RBPs, in an attempt to identify new RBPs with unique binding motifs. The method is based on characterizing the structural and electrostatic properties of the proteins. The electrostatic properties are mainly calculated from patches on the protein surfaces that are automatically extracted using our PatchFinderPlus algorithm [11],[23]. Combining an ensemble of features, we train an SVM system to distinguish RBPs from other non-nucleic-acid binding proteins that are characterized by large positive patches on their surfaces, with a very high accuracy of 88%. Applying a multiclass SVM, we show that we can successfully classify RBPs based on their RNA target (tRNA, rRNA, or mRNA), although we could not distinguish DBPs from RBPs. Interestingly, when tested on a nonredundant set of proteins that possess the RNA recognition motif (RRM), a typical RNA-binding motif known to be also involved in ssDNA binding and protein–protein interactions [24], we could successfully distinguish between RRM motifs involved in RNA-binding and the atypical RRMs involved in protein interactions.

## Results/Discussion

### Dataset Construction

The tremendous increase in structural information on RBPs enabled us to generate a nonredundant dataset of protein structures on which we were able to perform a comprehensive analysis. In the first step, we extracted from the Protein Data Bank (PDB) all RBP structures solved either by X-ray crystallography or by NMR. The original list was cleaned for redundancy by removing all structures that had more than 25% identity (for details see Materials and Methods). Further, the structures were annotated using the SCOP classification [25] and only protein chains including domains from unique families were retained in the final dataset. Overall, the final set included 76 nonredundant structures. As a control, we used a nonredundant database of 246 non-nucleic-acid binding protein chains (NNBP), used previously for nucleic-acid binding (NA-binding) prediction [11].

### Characteristic Features of RNA-Binding Proteins

#### The unique properties of the electrostatic surface patches

**Figure 1:**
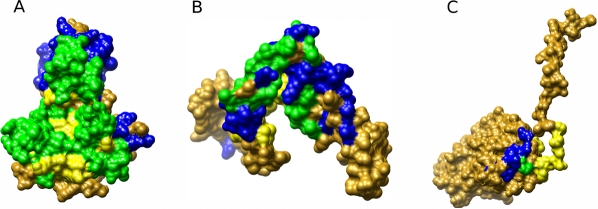
The overlap between the largest positive patch and the real RNA-binding interface in three different RBPs. (A) L1 ribosomal protein (1mzp), (B) rotavirus nonstructural protein (1knz), and (C) tymovirus coat and capsid binding protein (1ddl). The blue region represents the largest positive patch, yellow is the real binding interface (calculated as described in the Materials and Methods section), and green denotes the overlap between the extracted patch and the real interface. Notably, in (A) there is a large overlap (0.9) between the positive patch and the interface, while a very small overlap (0.05) was observed in (C).

RBPs bind RNA through a combination of structural modules [4]. Similar to DBPs, RBPs are known to bind RNA mostly via a positive electrostatic surface that complements the negative electrostatic charge of the RNA [5]. To detect new features that could be indicative of RNA binding, we extracted from each protein in our dataset the largest electrostatic patch on the protein surface using our PatchFinderPlus (PFplus) algorithm [23]. The Patch Finder algorithm was originally developed to automatically extract the largest positive patch from a protein surface [11]. Many studies have demonstrated the importance of electrostatic interactions in protein–DNA and protein–RNA recognition [22],[26],[27]. Previously it was shown that in DBPs, the largest positive patch of the protein encompasses, on average 80% of protein–DNA interface [11]. Interestingly, in the current study, we found that the overlap between the largest positive patch and the RNA–protein interface (interface was defined as described in the Materials and Methods section) varied dramatically between the different RBPs, ranging from 0% to 100% (Table S1). Figure 1 demonstrates the overlap between the largest positive patch and the real RNA-binding interface for three different RBPs. As exemplified in Figure 1A, in some proteins such as the L1 ribosomal proteins we found a very high overlap; whereas in other cases, for example, in the rotavirus non-structural protein and in the tymovirus coat protein shown in Figure 1B and 1C, respectively, the largest positive electrostatic patches did not coincide with the real binding interfaces. Overall, the average overlap was lower than the average overlap found previously for DBPs with a large standard deviation (68%±31% for RBPs).

The smaller overlap between the largest electrostatic patch and the experimentally verified RNA-binding interface suggests that in RBPs the interface may not always be a continuous patch, but rather several clusters of positive charged residues that are scattered on the protein surface. The large variation in the extent of the overlap between the positive patch and the interface may be related to the variability in the structural properties of the RNA. While DNA usually encompasses a relatively simple double helical structure, the three-dimensional structure of RNA is much more diverse, and could interact with the protein via independent regions that may not be continuous. For example, the tRNA synthetases usually bind the tRNA via two major regions, one region that binds the acceptor end of the tRNA and another region that binds the anticodon stem and loop region [28].

**Figure 2:**
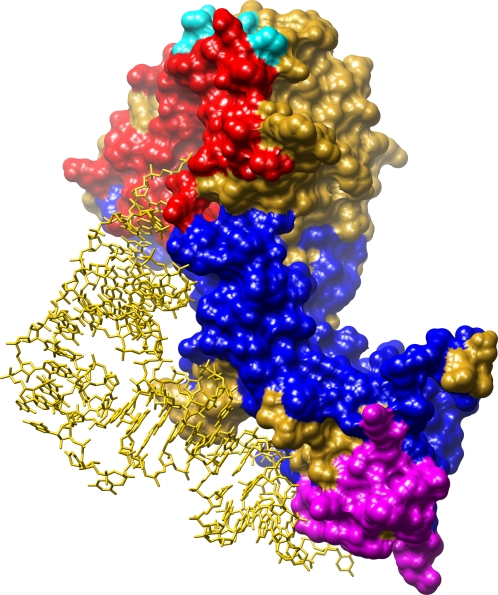
Illustration of the three positive electrostatic patches in the aspartyl tRNA synthetase (1asy). The largest patch is colored blue, the second largest patch is magenta, the third largest patch is cyan, and the negative patch is colored red. Interestingly, for the tRNA-binding proteins the protein binds via both the positive and the negative electrostatic patches.

In order to obtain a better representation of the RNA-binding interface, we analyzed the ten largest positive patches for each protein as well as the largest negative patch. The negative patch was defined as a continuous patch of grid points on the protein surface with an electrostatic potential of less than −2 *kT*/*e* (see Materials and Methods). Table S2 shows the average patch size and the percent overlap between the patch and the interface (relative to the interface and to the patch) for all 11 patches. Though on average the size of the largest positive patch was only double the average size of the negative patch, the overlap between the patch and the real binding interface (normalized to the size of the interface) was approximately five times larger for the largest positive patch compared to the largest negative patch. However in order to better represent the interface of RBPs, specifically for proteins with unique binding strategies such as the rotavirus protein shown in Figure 1B, we included in our analysis the three largest positive patches as well as the largest negative patch. Taken together these four patches cover on average 88% of the real interface, relative to 96% interface coverage when considering all 11 electrostatic patches (Table S2). Figure 2 illustrates the four different electrostatic patches on the surface of Aspartyl-tRNA Synthetase (PDB code: 1asy). As demonstrated, in the specific case of tRNA synthetase, it seems that the protein binds to the acceptor end of the tRNA close to the largest negative patch while the anticodon stem loop interacts with the second largest positive patch. It has been previously suggested that the positive patch in the center of the aminoacyl-tRNA synthetase has an important role in long range interactions, being the driving force for primary recognition [29]. It is important to note that in our method the electrostatic calculations were conducted on the monomer while most tRNA synthetases bind as dimers or tetramers, so the electrostatic properties of the biological binding interface may differ from the picture presented in Figure 2.

**Figure 3:**
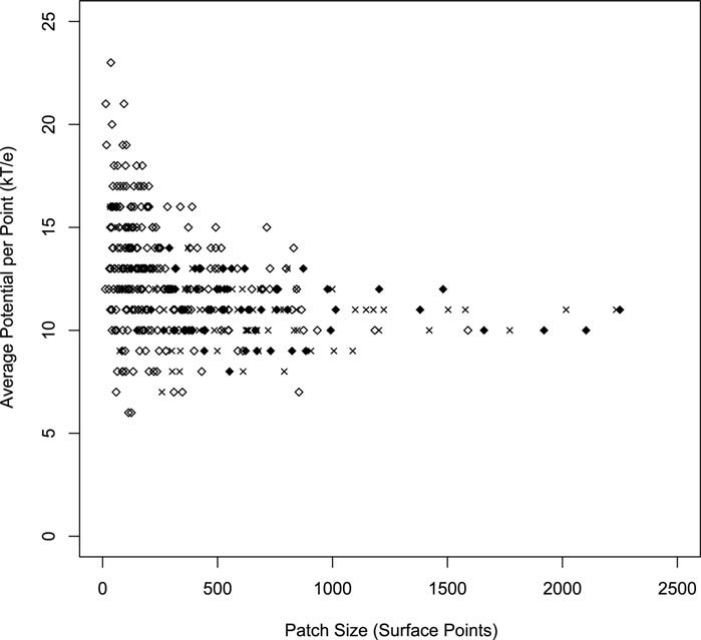
Patch size and surface potential of RBPs, DBPs, and NNBPs. Patch size is plotted against the average surface potential for all RBPs (black diamonds) compared to DNA-binding proteins (crosses) and non-NA binding proteins (open diamonds). As can be noticed, a large number of NNBP are characterized by relative large patch size.

As expected, we generally found that RBPs tend to have large positive patches on their surfaces. However, as was reported previously [11], many NNBPs also have large patches. Figure 3 represents the average potential and size of the largest positive patches in the data set of RBPs compared to DBPs and to a random set of NNBPs. The latter two datasets were extracted from Stawiski et al. [11]. In order to determine if the group of NNBPs with large patches differs from RNA-binding proteins by other properties, we sorted the control set of NNBPs based on the size of the largest positive patch and extracted an equal subset of 76 top-ranked NNBPs (see Materials and Methods). Our further analyses were conducted on three different datasets: RBPs, NNBP large-patch, and NNBP all. Among the features analyzed, we calculated 18 different structural and sequence features related to the largest positive patch, seven general protein features, four features related to the clefts on protein surface and the overlap between the clefts and the patch (a full list of the parameters and their description can be found in the Materials and Methods section). Averages and standard deviations were calculated for each feature in each subgroup. In addition, we applied standard statistic analyses (*T*-test and *F*-test) to test whether the averages and variances showed significant differences between the groups (Dataset S1). In addition we calculated the Spearman correlation coefficient for each parameter; the correlation coefficient (CC) values for the RBP vs. NNBP are shown in Figure S1. As demonstrated in the table and figure, when comparing the RBPs to the NNBPs, a number of parameters showed a significant difference. Among this set of features, the total clefts-patch overlap and the hydrogen bond potential donors showed the most significant difference between the groups (*p*-value for *T*-test 4.6E-28 and 2.7E-24, respectively). As expected, when comparing the patch features between the RBPs and the subset of the large patch NNBPs, the parameters related to the patch size were less able to distinguish between the two subsets. However other parameters of the patch, such as patch roughness and surface accessibility of the patch were among the most significant parameters (Dataset S1).

In order to examine whether other positive patches on the protein surface may contribute to characterize RNA-binding proteins, we calculated different features of the second and third patches and examined whether they differed between RBP and NNBP. Among these parameters, we included properties that are related to the patches themselves, such as the number of atoms in the patch, the average distance between the three positive patches and the distance between each of the positive patches and the negative patch. Overall, we extracted ten new parameters related to the additional patches (for details see the Materials and Methods section). The statistical analyses conducted on these ten parameters demonstrate that the sizes of the other positive patches as well as the largest negative patch were significantly different between the RBPs and NNBPs, with the largest differences observed for the third largest positive patch (Dataset S1). We found consistently that the size of the “other patches” was significantly different between the RBPs and the subset of large patch NNBPs. Surprisingly, we found that on average the size of the “other patches” including the negative patch was smaller in RBPs compared to the NNBP (Figure S1). Thus, though in unique cases, such as in tRNA-binding proteins, the secondary electrostatic patches (i.e., negative and second and third largest patches) may be involved in interactions with the RNA (either directly or indirectly via counter ions [30]), in the majority of RBPs the largest positive patch is the most significant electrostatic surface patch.

It has been shown previously that evolutionary information, i.e., the conservation of residues within the electrostatic patch, holds information on DNA-binding function and improves functional prediction [11],[27],[31]. However, evolutionary information may not be available when predicting novel structures. Furthermore, it has been claimed that adding evolutionary information to automatic predictions is time consuming [12],[13],[32]. Interestingly, in the current study, the conservation parameters of the patch were not found to be significantly different between RBPs and NNBPs, possibly due to the lack of informative evolutionary data available for the RBPs in our set. Nevertheless, in order to speed up the method and allow for identification of novel structures, we did not include evolutionary information within our feature set. The fact that the current method does not rely on any type of evolutionary information makes it distinctive from all other available methods for predicting nucleic acid binding properties from structure (e.g., [11]).

#### Global protein features

In addition to the features extracted from the surfaces patches alone, we calculated other global parameters of each protein, such as the molecular weight of the protein, the protein's surface accessibility, the size of the largest clefts on the protein's surface, and the overlap between the clefts and the patches. Among the general properties, the molecular weight and surface accessibility were significantly lower in the RBPs compared to NNBPs, both when considering the full set of NNBPs as well as when analyzing the subset of NNBPs with large positive patches (Dataset S1). As described above, the most significant parameter (when considering the full control set) was the percent overlap between the largest clefts of the protein surface and the largest positive patch, which was clearly higher in RBPs. Although the overlap between the surface clefts and the electrostatic patch was not the most significant parameter when comparing the RBPs to the NNBPs with large patches, it was still found to be significantly higher in the RBPs (*p*-value for *T*-test 6E-4).

In a previous study, Ahmad and Sarai observed a higher electric moment in DBPs relative to other non-DNA binding proteins [33]. Recently, Fedler et al. [34] have shown that a high moment dipole is characteristic of all nucleic acid binding proteins, including ribosomal proteins. We calculated the dipole and quadrupole moments for all the proteins in our dataset. As expected, the dipole moment was significantly higher in the RBPs compared to NNBPs. When comparing the dipole moment between the RBPs and the NNBPs with large patches, only the *F*-test showed a highly significant difference.

### Classifying RNA-Binding Proteins Using a Support Vector Machine

In order to examine whether the calculated features can be used to distinguish the RBPs from other proteins (specifically NNBPs that possess large positive patches), we applied a machine learning approach, namely, the support vector machine (SVM). SVMs are supervised learning methods; they take as inputs a set of features, called feature vectors, to train a model and output a classification for a query based on the model. After being trained on a set of feature vectors whose expected outputs were already known, SVMs are able to classify new input vectors. Recently, SVMs have been increasingly used in addressing the problems of protein classification, including fold recognition [35] protein structural class prediction [36], protein–protein interaction [37], membrane protein type recognition [38],[39], and G-protein coupled receptors classification [40]. Furthermore, SVMs have been utilized to solve protein classification problems and were shown to complement other methods that are based on sequence similarity [41].

**Figure 4:**
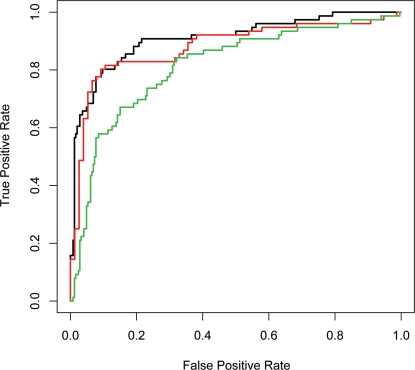
ROC plots illustrating the SVM results for RBPs classification. In black, RNA-binding proteins versus non-NA-binding proteins (AUC = 0.90); in red, RNA-binding proteins versus non-NA-binding proteins with large patches (AUC = 0.88); in green, RNA-binding proteins versus non-NA-binding proteins when including only the electrostatic patch properties (AUC = 0.81).

We applied an SVM classifier to distinguish between the nonredundant set of RBPs and the NNBPs, as well as between the RBPs and the subset of NNBPs with large positive patches. For training, we applied a normalized feature vector that included all 40 sequence and structural parameters that were extracted from both the electrostatic patches and from the whole protein. For testing, we applied a cross-validation (leave one out) test, where for each SVM run, one protein was extracted from the training and tested separately. To evaluate the SVM performance, we plotted the ROC curve (receiver operating characteristic) describing the relationship between the false positive rate (FPR) and the true positive rate (TPR). The results of the SVM test are illustrated in Figure 4; overall we could successfully distinguish RBPs from NNBPs and from the subset of large-patch NNBPs with 88% and 86% accuracy, respectively (details in Table 1). The areas under the curve (AUCs) calculated for these experiments were 0.9 and 0.88, for the full and subset, respectively. The high performance achieved for distinguishing RBPs from other protein with large patches is extremely encouraging, since by visual inspection of the physical and electrostatic properties of the proteins one cannot distinguish between the two functionally different groups. Furthermore, when calculating each parameter independently, many of the properties did not show significant differences between the RBPs and NNBPs with large positive patches; only by combining all parameters using an SVM could we clearly distinguish between the groups. These results imply that RBPs have unique properties that can distinguish them from proteins that do not bind nucleic acids. Importantly, the distinctive properties do not relate either to the fold of the protein or to its binding motif.

**Table 1:** Summary of SVM results for different classifiers.

	FP	FN	TP	TN	AUC	Sensitivity (%)	Specificity (%)	Accuracy (%)	MCC
RBPs vs. NNBPs 40 parameters	24	16	60	222	0.90	80	90	88	0.67
RBPs vs. NNBPs 10 to parameters	31	14	44	215	0.90	76	87	80	0.54
RBPs vs. NNBPs electrostatics features only	99	11	65	147	0.81	86	60	66	0.38
RBPs vs. NNBPs large patches	7	15	61	69	0.88	80	91	86	0.72
RBPs vs. DBPs	29	36	40	29	0.51	53	50	51	0.03

The table summarizes the SVM results for four different classifiers: RBPs vs. NNBPs (40 parameters), RBPs vs. NNBPs (10 top parameters), RBPs vs. NNBPs including only electrostatic patch features (34 parameters), RBP vs. large-patch NNBPs, and RBP vs. DBP.

TP, true positives; TN, true negatives; FP, false positive; FN, false negatives; AUC, area under the curve. Sensitivity, specificity, accuracy, and MCC (Matthew's correlation coefficient) were calculated as described in Materials and Methods section.

To ensure that the good performance of the cross-validation test was not due to overfitting of the data, we tested an independent set of hypothetical proteins from the PDB database, which were solved by structural genomics projects and classified as RNA-binding proteins. To prevent circularity, the hypothetical proteins chosen for the test did not share more than 25% identity with any of the proteins in our training set, each representing a different fold and a different RNA-binding motif. Furthermore, since in many cases RNA binding is automatically predicted based on the existence of a known RNA-binding motif or sequence similarity, we included in the testing set only proteins that were verified experimentally to bind RNA (detailed description of the test set is given in Table S3). Overall we tested 13 proteins verified experimentally to bind RNA and 10 (78%) were successfully predicted as RBPs. Interestingly, all three false negative results were annotated to be involved in tRNA binding.

### RNA-Binding Proteins vs. DNA-Binding Proteins

Since RBPs share many common characteristics with DBPs in terms of their electrostatics and structural features, clearly the most challenging goal would be to distinguish between these two groups. Several studies have demonstrated that RNA-protein recognition differs from DNA recognition in several aspects [22],[42],[43]. Since the RNA and the DNA adopt different helical parameters, dsDNA usually adopting a B-form while dsRNA adopts A-form helices frequently interrupted by internal loops and bulges [44], it is expected that the electrostatic patches will differ between the two types of NA binding proteins. As a first step we examined whether the new feature set selected for predicting RBPs would be as efficient for predicting DBPs. To test this, we calculated the 40 features for the set of nonredundant DNA binding proteins and built an SVM classifier for DBPs vs. NNBP. As for the RBP classifier, here too we tested the DBPs against the set of nonredundant NNBPs applied in Stawiski et al. [11],[45]. Overall the SVM performed similarly to the RBP vs. NNBP classifier, though with lower accuracy (85%). Interestingly, the current SVM results were slightly inferior to those previously reported with artificial neural network (ANN) classifiers [11]. These results are as expected, since the feature set we used in the current study was specifically designed for predicting RBPs and excluded the evolutionary information. Nevertheless, the relatively high performance achieved for predicting DBPs reinforces that the two sets of NA binding proteins have much in common. Next, we examined how well the SVM classifier discriminates between RBP and DBPs. Using the set of 40 features we were not able to distinguish RBPs from DBPs (Table 1).

It is well established that certain RNA-binding motifs can also bind DNA and vice versa (e.g., [46]). Furthermore, it is anticipated that nucleic-acid binding proteins have several roles in gene expression pathways and thus potentially have the intrinsic ability to bind both DNA and RNA [47]. Nevertheless, after excluding from our training data all proteins that bind via motifs known to bind both DNA and RNA (e.g., C2H2 zinc finger) and generating two unique data sets, single strand RBPs (ssRBPs) vs. double stranded DBPs (dsDBPs), we still could not distinguish between the RPBs and DBPs based on the above parameters. When testing on 36 dsDNA vs. 40 ssRNA-binding proteins (full list given in the Materials and Methods section), we classified only 19 as DNA-binding and 21 as RNA-binding, achieving a weak overall accuracy of 47%. This suggests that further refinement of nucleic-acid binding function will be required in order to build a classifier to distinguish exclusively RNA-binders from DNA binding proteins.

### Feature Selection

To further study the role of the electrostatic properties in discriminating RBSs from NNBPs we excluded from the SVM classifier all features related to the protein parameter group (features 19–25 in Dataset S1). Though the SVM performance was evidently reduced upon eliminating these features (Table 1 and Figure 4), we still found that the electrostatic features were sufficient for distinguishing RBPs from NNBPs. Further, to test which of the calculated features contributes most to the RNA-binding prediction, we performed a Recursive Feature Elimination procedure (RFE) (see Materials and Methods). When applying the RFE algorithm to our data, eliminating 50% of the features at each iteration, for the first three rounds of selection we did not observe notable changes in the AUC value. Only in the fourth iteration did the SVM performance decrease dramatically. The lists of the selected features that were retained in the third iteration (both when testing RBPs vs. all proteins and the RBPs versus NNBPs with large-patches) are shown in Table 2. As expected, the majority of features (8/10) selected among the top ten properties in the RBPs vs. NNBPs classifier were electrostatic-related features. Interestingly, there was a large overlap between the top ten parameters that were selected with the RFE algorithm in both classifiers. These results reinforce that the differences between the RBPs and the NNBPs are related to the function of the RBP and not simply to the size of the patch.

**Table 2:** Summary of the discriminating features selected by SVM-RFE.

Feature	RNA-binding vs. non-RNA-binding		RNA-binding vs. non-RNA-binding largest patches	
	*F*-test	*T*-test	*T*-test	*F*-test
Molecular weight	**2.7E-07**	8.5E-02	**9.8E-08**	8.8E-01
Protein surface accessibility	**4.9E-03**	8.2E-01	**5.2E-05**	**2.7E-09**
Patch potential	**5.5E-15**	**8.5E-15**	2.2E-01	**2.3E-07**
Patch surface accessibility	**1.9E-21**	**1.9E-13**	3.0E-01	**2.7E-09**
Quadrupole	2.5E-02	**1.7E-07**	NA	NA
Dipole	**2.0E-10**	**1.5E-31**	8.5E-02	**5.5E-12**
Patch size	**1.3E-19**	**1.8E-17**	1.9E-01	**1.0E-08**
Number of atoms in largest positive patch	**8.6E-08**	**1.8E-03**	4.8E-01	6.6E-02
Patch surface overlap	**4.6E-28**	**5.7E-09**	**6.1E-04**	**5.8E-05**
Number of atoms in the negative patch	**4.4E-03**	**7.1E-11**	**3.4E-04**	**1.0E-07**
Size of largest cleft	NA	NA	1.5E-02	6.4E-01

*P*-values are given for the *T* and *F* statistics for the two different classifiers: RBPs vs. NNBP (left) and RBPs vs. NNBPs with large patch (right). Bold numbers represent statistically significant results, where the Bonferroni correction was applied for multiple testing. NA denotes that the parameter was not selected by the RFE procedure for the specific classifier, and thus statistical analysis was not applied.

To further test the contribution of each one of the top ten parameters to the final SVM performance we conducted a backwards feature selection procedure and eliminated, in turn, each one of the parameters from the feature set and repeated the SVM testing (using the same cross-validation approach). For each test, we calculated the ΔAUC, which is the difference between the AUC achieved when including the feature and the AUC after excluding the feature. When testing on the full dataset of RBPs vs. NNBPs, no notable reduction was observed after eliminating a single parameter from the top ten list. Generally, the ΔAUC analysis suggests that all features that were selected by the RFE contribute equally to the SVM performance. Nevertheless, as shown in Table 1, when including only the top ten features in the RBP vs. NNBP classifier, the SVM achieved the same results as with the full parameter set. However, in the more challenging case of RBPs vs. the large patch NNBP set, all 40 features were needed to achieve the best performance (both in terms of sensitivity and selectivity). Thus for achieving the best performance for RNA-binding classification in general, we consistently use the extended classifier.

### Independent Testing on an RNA-Binding Motif: The RRM as a Test Case

Although the 76 RBPs in our positive set were cleaned for redundancy both at the sequence and structural (family) level, within the structural groups we still had representatives of RBPs with a common binding motif (e.g., two proteins with an RRM motif). In order to be confident that the SVM results do not depend on having several proteins sharing the same binding motif within our dataset, we applied a motif-independent test. In this test we withheld, in turn, all proteins sharing a common binding motif and trained the SVM on the remaining proteins (Table S4). We then tested each member of the binding motif family on an SVM classifier from which that group had been completely withheld. As shown, the motif test performed exactly the same as the original test did, with very slight differences in the discriminating values obtained for each tested protein (Table S5). Interestingly, there was one motif group of tRNA-binding proteins which was completely misclassified (seven out of seven proteins) using both the RBP classifiers (leave-one-out vs. leave-family-out).

Overall the SVM results suggested that in the majority of cases RBPs can be uniquely characterized, independent of their binding motif. These results encouraged us to further test whether our method could discriminate RNA from non-RNA-binding proteins that possess a common binding motif. The RRM is one of the most abundant protein domains in eukaryotes. This motif is a classical RNA-binding motif, however it has been found to appear in a few ssDBPs, and most interestingly, in many proteins the RRM motif is involved in protein-protein interactions [24]. While the RRMs that mediate protein interactions commonly interact both with RNA and protein (frequently the protein-protein interactions are between two RRMs), in unique cases the RRM is solely involved in protein–protein interactions [24]. To test whether our method can distinguish between these cases, we obtained from the PDB a nonredundant set of protein chains that possess an RRM domain (Table S6). The structures were extracted automatically from PDB using a 35% sequence identity cutoff. The existence of the RRM motif was further verified against the pfam database [48]. Further, we tested each of the 27 protein chains with our SVM classifier using all 40 features. Consistent with the motif-independent test, the proteins were tested against a classifier in which the two original proteins including an RRM were excluded from the training. Overall, amongst the 27 protein chains, 21 were classified as RBPs, with one marginal prediction and six chains classified as NNBPs (Table S6).

Amid the six protein chains that were classified as NNBPs was the RRM domain of Y14 from the Y14-Magoh complex (PDB code: 1rk8A), which has been confirmed experimentally to be involved only in protein-protein interactions [24],[49]. In addition, the RRM1 domain of the SET1 histone methyltransferase (PDB code: 2j8aA) was classified as NNBP. The latter result is consistent with experimental studies which have shown that the RRM1 of the SET1 protein does not bind RNA in vitro, suggesting that the protein may be involved in RNA binding in vivo only via RRM–RRM interactions [50]. Three other chains that were predicted as NNBPs are the RRM of U2AF 35 (PDB code: 1jmtA) and the atypical RRMs (U2AF-homology motif) of U2AF65 and SFP45 (PDB codes: 1opiA and 2pe8A, respectively); all three were confirmed to be involved in protein–protein interactions in the spliceosome [51]. Interestingly the protein chain of the splicing factor SRp20, including an RRM and a TAP binding motif (PDB code: 2i2yA), was also classified as NNBP. It is plausible that these results are influenced by the existence of the TAP protein binding domain within the protein chain [52]. Notably, among the chains classified as RBPs, only in the case of elF3 (PDB code: 2nlwA) was our classification in contradiction to the experimental data, which suggests that the RRM motif does not bind RNA directly [53]. The elFj is part of a large multiprotein complex involved in initiation of translation in eukaryotes, binding the 40s ribosomal subunit. Recent studies have shown that the RRM of elFj interacts with elFb, which directly binds the ribosome [53]. Interestingly, we found the largest positive patch of the surface of elFj is on the opposite side of the RRM (data not shown), suggesting that the protein might not be interacting with the rRNA via the RRM. Consistent with our previous result, the RRMs of UP1, which binds RNA and ssDNA, was classified as RNA binding.

**Figure 5:**
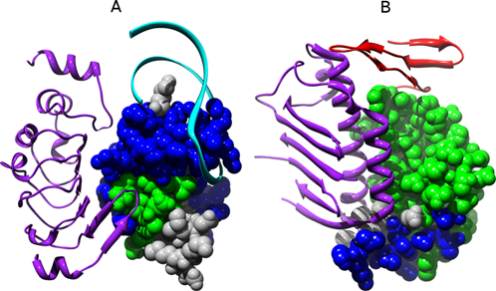
The largest electrostatic patch mapped on the protein structure of two RRM domains. (A) The U2 snRNP A′ from the U2B″–U2A′ complex (1a9nA) and (B) the Y14 protein from the Y14-Magoh complex (1rk8A). Blue represents the largest electrostatic patch and green the RRM motif as defined by pfam. For the RNA-binding RRM domain the largest electrostatic patch overlaps the RNA-binding interface, while no overlap is observed between the largest electrostatic patch and the protein-protein interface of the Y14 protein. Notably, the largest positive patch is much smaller in the latter case.

Overall, our results suggest that we can distinguish between RRM motifs involved in nucleic acid binding from those that are involved in protein–protein interactions. However, since our current method can only distinguish RNA from non-NA binding, in the ambiguous cases where the protein is involved in both RNA and protein interactions (either via the RRM motif or another motif), the SVM results may not be sufficient for prediction. To better understand which of the features used for the SVM training contributed to the ability of the classifier to distinguish the RNA from non-RNA-binding RRMs, we split the data into positive and negative predictions and applied the Mann–Whitney–Wilcoxon test on each one of the 40 parameters. Interestingly, the features that showed the most significant differences between the positive and negative groups were the features related to the electrostatic patches (Table S7). Figure 5 illustrates the largest positive patch in the U2B″–U2A′ complex (PDB code: 1a9nA), including an RRM known to be involved both in RNA and protein interactions, in comparison to the largest electrostatic patch in the Y14 proteins (PDB code: 1rk8A), including an RRM motif which is involved only in protein-protein interactions. In the U2B″–U2A′ complex, the large positive patch (blue) overlaps the RRM (green), which interacts directly with the RNA, while in the Y14 complex the largest positive patch is relatively small and does not overlap with the RRM motif, which is involved in the interaction with the Magoh protein.

### The Unique Properties of tRNA-binding Proteins

A critical step in evaluating the strength of a classifier is to carefully examine the cases were it fails (i.e., the false negatives and the false positives). As mentioned earlier, when we analyzed the results of the SVM, we discovered that amongst the false negative results there were several tRNA-binding proteins. Previous structure analysis of the aminoacyl-tRNA synthetases demonstrated that these proteins bind tRNA via multiple domains, each of which independently recognizes different sites on the RNA [54]. In addition, it has been observed that the aminoacyl tRNA synthetases possess an unexpectedly negatively charged surface [29]. Other RBPs, such as the bacterial release factors that mimic tRNA also have highly negatively charged surfaces [55]. To further explore the unique properties of tRNA-binding proteins, we generated a set of 13 nonredundant tRNA-binding proteins that share not more than 25% sequence identity among them (six of them were in our original dataset). Further, we built a new SVM classifier for the 13 tRNA-binding proteins against all RBPs (excluding the tRNA-binding proteins). Applying a cross validation test, the SVM was able to separate the two data sets with very high accuracy (AUC = 0.94). Interestingly, when testing the misclassified proteins from the hypothetical test (Table S3) against the tRNA vs. RBPs classifier, all three proteins were classified correctly as tRNA-binding. These results are consistent with previous studies on tRNA-binding proteins that showed a very different mode of binding to RNA relative to other RNA-binding proteins [56], and are also consistent with recent sequence-based RNA-binding predictions, which demonstrated high prediction accuracy for tRNA-binding proteins [17],[18].

**Figure 6:**
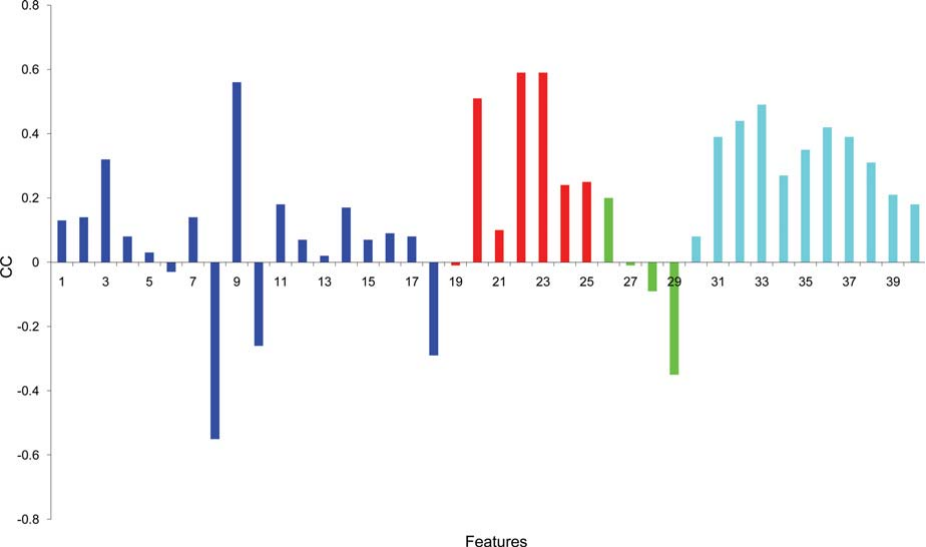
Spearman correlation coefficient values (ρ) calculated for each one of the 40 features comparing tRNA vs. all RBPs. The features are colored by group ( detailed numbers are given in Dataset S1): Dark blue represent features related to the largest positive patch, in red are features related to the whole protein, in green are cleft-patch related features, and in cyan are the “other patches” features. The protein feature and the features related to the secondary electrostatic patches showed the highest CC with a positive sign, denoting that these features are greater in the tRNA group.

To test which are the most significant features for distinguishing between the tRNA-binding proteins and all other RBPs, we calculated the Spearman correlation coefficient (CC) of each one of the 40 features. Figure 6 demonstrates the correlation values (ρ) for the 40 features (numbered as in Dataset S1). Interestingly, the features that showed the highest correlations were the molecular weight and surface accessibility of the whole protein (colored in red); both were significantly higher in the tRNA group (*p*∼10^−16^), suggesting that tRNA-binding proteins are generally larger than other RBPs in our data. In addition, the roughness of the large positive patch was significantly greater in the tRNA group, while the average surface accessibility was lower in the group of tRNA binders compared to other RBPs. Strikingly, as can be noticed on the right hand side (blue bars) of Figure 6, all the ten features related to the “other patches” (i.e., the size of the negative, second and third patch, distances between the patches, etc.) were among the top ranked features that showed a significant, high CC. These results emphasize that the tRNA-binding proteins have unique electrostatic properties that can be utilized for identifying novel proteins possibly involved in tRNA processing. Moreover, we noticed that the electrostatic properties distinguishing between the tRNA and the other RBPs are mainly related to the secondary patches and not to the largest positive patch.

### Multiclass SVM

**Figure 7:**
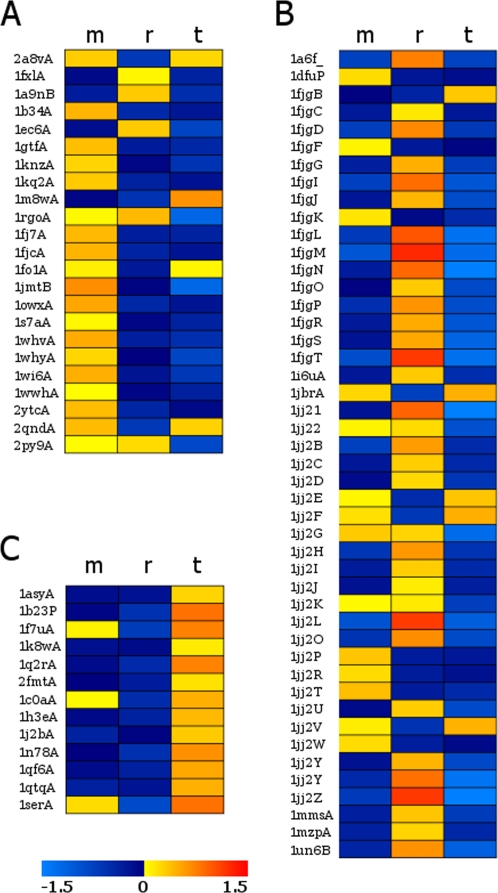
Multiclass SVM analysis for 3 subgroups. (A) mRNA, (B) rRNA, and (C) tRNA. Each protein in each of the subgroups was tested against the three different classifiers. For each subgroup, the SVM results for the mRNA classifier are shown in the most left column, results for the rRNA classifier in the middle column, and for the tRNA classifier in the right column. SVM results are color-coded: red representing high positive results and shaded blue representing low negative results (see color bar).

Following these observations, we were encouraged to test whether we could automatically distinguish between different RNA-binding strategies of known RNA-binding proteins. Previously, a multi-SVM approach was applied for classifying genes involved in different stages of the gene-expression pathway into subclasses based on microarray data [47],[56]. To test whether a multiclass approach could be applied for classifying subsets of RBPs based on the type of RNA they bind, we built three new SVM classifiers, which were trained on experimentally verified RBPs: an rRNA-binding protein classifier, an mRNA-binding protein classifier and a tRNA-binding protein classifier (see Materials and Methods). It is important to note that the groups were not split based on the RNA-binding motif and in several cases the same motif (such as the KH motif or the zinc finger motif) was found in different subsets. The 82 RBPs were tested subsequently on each of the three classifiers (in each case, the tested protein was held out from the training set). Finally, a protein was assigned a value based on the classifier in which it achieved the highest positive discriminating value. The results of the multi-SVM test are shown in Figure 7 and summarized in Table 3 (detailed results are given in Table S8). As demonstrated in Table 3, in all three subclasses the highest number of proteins was correctly assigned to the appropriate subgroup. As expected, the best results were obtained for the tRNA-binding proteins, where 13 of the 13 tRNA-binding proteins were clearly assigned as tRNA-binding. As can be observed in Figure 7C, the majority of tRNA-binding proteins also achieved a positive score in the mRNA classifier, though in all cases the scores were lower than for the tRNA classifier. Different studies have demonstrated that tRNA synthetases are also involved in mRNA-binding; for example, it was recently shown that the Glu-Pro tRNA synthetase has a role in blocking the synthesis of specific proteins by binding to the 3′ UTR of their mRNA [57]. In the rRNA-binding protein group, while the majority of the proteins (70%) scored the highest in the correct rRNA classifier, some proteins were still misclassified. Among the 14 misclassified proteins, nine were classified as mRNA and five as tRNA (Figure 7B and Table S8). These results are consistent with the notion that ribosomal proteins have several other functions in the gene expression pathway [58]. Interestingly, included in the set of rRNA proteins that were misclassified as tRNA, was the ribotoxin restrictocin bound to the sarcin/ricin domain (SRD) from the large ribosome subunit (PDB code 1jbr). This toxin disrupts elongation factor binding to the SRD domain that also binds tRNA [59]. Notably, our classification is purely based on structural information and does not rely on homology information, and thus it is expected to achieve lower performance compared to available sequence-based rRNA classification [17].

**Table 3:** A table summarizing the multi-SVM results for 3 subclasses of RNA-binding proteins: tRNA, rRNA, and mRNA.

	Predicted as mRNA	Predicted as rRNA	Predicted as tRNA	Total
tRNA	0	0	**13**	**13**
rRNA	9	**32**	5	**46**
mRNA	**17**	5	1	**23**

Bold numbers represent the classifier in which the majority of proteins were predicted. As can be noticed by the diagonal the majority of predictions were assigned to the correct subclass.

Finally, for the mRNA group we collected 23 nonredundant proteins: 13 proteins that bind mRNA at the different stages of the gene expression pathway (transcription, splicing, polyadenylation, etc.) and ten other proteins that bind mRNA such as hydrolases, export factors, viral mRNA, binding, etc. (for details see Table S8). Overall, amongst the 23 mRNA-binding proteins composed of different binding motifs, 73% of the proteins were assigned correctly (Figure 7A). Among the false negatives, five were predicted as rRNA. Notably, the false negative mRNA-binding proteins did not belong to a certain binding motif or fold (2 KH, 1 RRM, 1 LRR, 1 PUF, and 1 Zinc Finger), again reinforcing that our classification is motif-independent.

### Electrostatic Patch and RNA-Binding Interface

**Figure 8:**
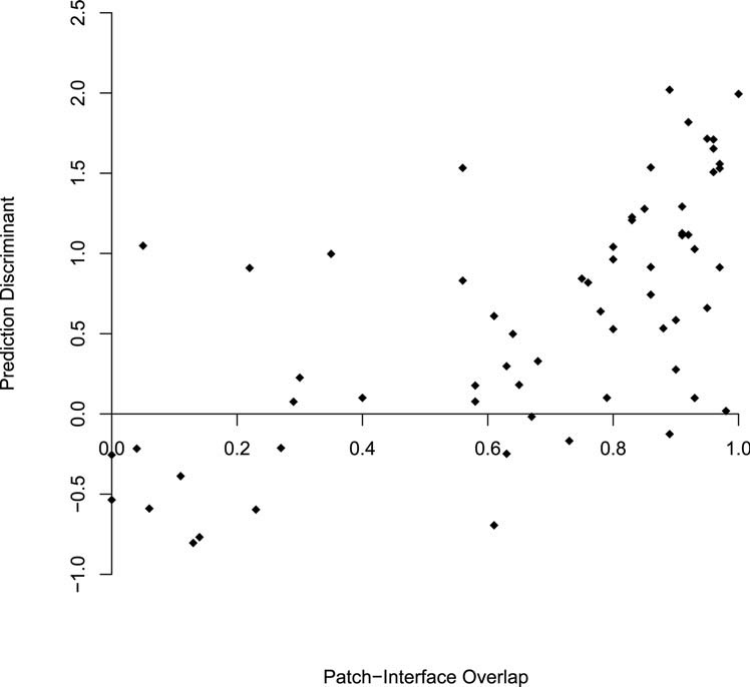
The correlation between the patch-interface overlap and the discriminate value obtained from the SVM classifier. As illustrated, the prediction power of the algorithm depends on the success in identification of the functional interface.

As noted, the basic assumption behind our algorithm was that the electrostatic patch is related to the nucleic acid binding interface. Thus it is expected that the success of the method would depend on the correlation between the patch residues (identified automatically by our algorithm) and the experimentally defined RNA-binding interfaces. We previously found that in DNA binding proteins the largest positive patch of the protein encompasses, on average 80% of the protein-DNA interface [11]. As demonstrated in Figure 1, the positive patch of the RBPs does not always coincide with the real binding interface. Here we tested the correlation between the patch–interface overlap and the confidence of the RNA-binding classification, as derived from the SVM. Applying an SVM, each tested protein was assigned a discriminating value (generally the distance of the protein from the hyper plane). As illustrated in Figure 8, when applying a Spearman correlation coefficient, we found a significant positive correlation (ρ = 0.64, *p*<10^−8^) between the percent overlap of the positive electrostatic patch and RBP interface and the discriminating value obtained by the SVM. These results imply that the success of the method at classifying RBPs from NNBP strongly relies on the degree of overlap between the largest positive patch and the binding interface. The correlation between the patch-interface overlap and the SVM performance is also consistent with the feature selection results that showed that the majority of the features contributing to the performance were associated with the largest positive patch.

### Conclusions

In this study we applied a machine learning approach to classify RNA-binding function from the 3D structure of the protein. Using features extracted from the positive electrostatic patches on RNA and non-nucleic-acid binding proteins, we trained an SVM to classify RBPs. We show that our method successfully distinguishes, with relatively high accuracy (88%), the RBPs from other proteins that do not bind nucleic acids. Similar results were achieved both when applying a cross-validation (leave one out) approach and when testing an independent set of proteins solved by a structural genomics initiative and confirmed experimentally to bind RNA. However, our method was not able to distinguish between RNA and DNA binding proteins. Interestingly, although the RBPs were distinguished from non-nucleic acid binding proteins by a combination of properties, we show that the success of the classification strongly depends on the degree of overlap between the largest positive patch and the real binding interface. Furthermore, we could show that the results do not depend on the RNA-binding motif, and correct classification was also achieved when we withheld all proteins that share a similar binding motif. Overall, our method is applicable for classifying RBPs that are generally very diverse in terms of their structure, function, and RNA recognition motifs. Moreover, since the method does not rely on sequence or structure conservation, we suggest that it could be applied to identify novel nucleic acid binding proteins with unique binding motifs.

One of the great challenges in classifying ligand binding proteins (such as RBPs) is to be able to identify to which ligand it will bind. For this purpose, we have applied a multiclass SVM classifier, which was trained on three different groups of known RBPs classified according to their RNA target: tRNA, rRNA, or mRNA. In the majority of cases, given that a protein is a RBP, we could assign it to a specific subgroup. Consistent with sequence-based predictions, we succeeded in correctly predicting all tRNA-binding proteins, whereas only 70–73% of rRNA and mRNA-binding proteins were assigned correctly. Overall, the results we obtained are very encouraging, reinforcing the idea that structural properties of proteins that are not directly related to the protein fold can give clues to the protein's interacting partner. It is important to note that subclassification of the RBPs to the three subgroups (mRNA, rRNA, or tRNA) using our multiclass approach is only possible given the prior knowledge that the protein binds RNA. Finally, consistent with other recent studies, our results suggest that electrostatic features of the protein surface can contribute to fine-tuning predictions of nucleic-acid binding proteins.

## Materials and Methods

### Dataset Construction

A nonredundant set of RBPs was constructed based on the RNA recognition motifs definition in Chen and Varani [5]. Additional proteins have been added to the data set based on manual data mining of the RCSB Protein Data Bank using the SCOP family definition [60]. From each SCOP family, only one representative protein was added to the dataset. From each protein included in our dataset, only the chain or chains containing the RNA-binding domain were analyzed. The chains involved in RNA binding were selected by manual inspection using the PyMOL viewer [61]. All selected chains were further cleaned for redundancy, including only proteins that share less than 25% sequence identity. In addition, the PISCES program [62] was applied to automatically select for proteins with resolution better than 3.5 Å, *R*-factor ≤0.3, and a sequence length from 40 to 1000 amino acids.

The NNBP data set was constructed from Hobohm and Sander's “pdb select” list of proteins [63] used previously in Stawiski et al. [11], excluding all proteins involved in binding NAs. Similarly to the RBP set, the control data set was further cleaned by excluding sequences with more than 25% identity. The subset of large-patch NNBPs was selected from the control set by sorting the proteins by the size of the largest patch; the top 76 proteins were chosen: 1skf, 1a6oA, 1pbe, 1a17, 1hcl, 1a7s, 1oaa, 1gox, 1ayl, 1uae, 1oyc, 1fnc, 1hcz, 1cpt, 1pda, 1lam, 1frb, 1ido, 1drw, 1fds, 1axn, 1gky, 1opr, 1lfo, 1ciy, 1fmk, 1csn, 1nsj, 1ndh, 1a8p, 1atg, 1bg2, 1csh, 1lit, 1rcb, 1cot, 1lid, 1bdb, 1fit, 1pbv, 1br9, 1ppn, 1a53, 1czj, 1a8e, 1mai, 1dhr, 1lki, 1c52, 1mrp, 1sbp, 1php, 1gnd, 1nfp, 1af7, 1aj2, 1alu, 1rhs, 1ddt, 1amf, 1ng1, 1al3, 1koe, 1mla, 1bhp, 1lbu, 1kte, 1nox, 1amm, 1a6m, 1phd, 1gen, 1b6a, 1gsa, 1ash, 1moq

A nonredundant set of RBPs that bind ssRNA was constructed from the original dataset and includes the following 40 protein chains: 1a1tA, 1a9nB, 1aq3A, 1asyA, 1b23P, 1b34A, 1cx0A, 1ddlA, 1e8Ob, 1ec6A, 1f7uA, 1fjgB, 1fjgC, 1fjgF, 1fjgG, 1fjgI, 1fjgJ, 1fjgK, 1fjgL, 1fjgM, 1fjgN, 1fjgO, 1fjgP, 1fjgR, 1fjgS, 1fjgT, 1gtfA, 1h2cA, 1hq1A, 1i6uA, 1jidA, 1k8wA, 1knzA, 1kq2A, 1m8wA, 1mmsA, 1mzpA, 1rgoA, 1ropA, 2fmtA. The set of dsDNA binding proteins was selected from the DNA binding proteins dataset [11]. The 36 selected protein chains were: 1a02F, 1a31A, 1a3qA, 1a73A, 1aayA, 1am9A, 1b3tA, 1bdtA, 1bnkA, 1cktA, 1cmaA, 1d66A, 1ddnA, 1ecrA, 1fokA, 1hmiA, 1ignA, 1ihfA, 1lmb3, 1mnmA, 1pdnC, 1pnrA, 1sknP, 1tc3C, 1trrA, 1tupA, 1wetA, 1xbrA, 2bopA, 2dgcA, 2hmiA, 2irfG, 2nllA, 3croL 3mhtA, 3pviA

For the independent test set we extracted from PDB RNA-binding proteins that were classified as “hypothetical” or “structure genomics.” The RNA-binding function was defined based on Gene Ontology (GO) terms, considering the molecular function level http://www.geneontology.org/. In cases where GO annotation was not available, we included proteins that were defined as RNA-binding proteins in the primary citation. Further, the list was manually curated, including only proteins that were verified experimentally (based on the literature) to bind RNA. Importantly, proteins which were defined by GO as RBP based on the existence of an RNA-binding domain or on high sequence similarity to a known RBP were not included in the final list. The detailed list of the hypothetical proteins is given in Table S3.

### Feature Calculations

Overall, 40 different input features were calculated; the features can be roughly classified into four major subgroups:

- **Largest patch parameters** including the patch size and potential number of atoms/ residues in patch, percent of α/β/loop in patch, patch surface accessibly, average surface accessibility per residue, patch roughness, number of Lys, Arg, overall polar amino acids in patch, potential hydrogen bond acceptors/donors in patch, satisfied acceptors/donors in patch, percent hydrogen bond in patch.
- **Protein parameters** including molecular weight and molecular weight per residue, radius of gyration/normalized radius of gyration, protein surface accessibility, dipole, and quadrupole moment.
- **Cleft/patch parameters** including the overlap between the largest, second largest, and third largest clefts, and largest patch, as well as the overlap between all three clefts and the largest electrostatic patch.
- **Parameters related to other surface patches** including number of residues in the lysine out patch [11] and in the negative patch, number of atoms in the second and third largest patch, number of atoms in the negative patch, distance from the largest positive patch to the second and third largest positive patches, and distances from the largest negative patch to the largest, second largest and third largest positive patches.

The PatchFinder algorithm[11] was applied to extract all continuous positive patches on the proteins surface with a cutoff of >2*kT*/*e* [23]. The patches were sorted based on the number of grid points included within the patch, and the largest three patches were selected. The largest negative patch (<−2*kT*/*e*) was extracted as described in Stawiski et al. [11]. The distances between the patches were calculated from the center of mass of each patch. Protein features were calculated as described in [11]. In addition, the dipole and quadrupole moments were calculated using the Protein Dipole Moments Server [64]. Interface residues were calculated using the Intervor web server [65]. Intervor calculated macromolecular interface using the Voronoi cells approach. This approach was shown to be highly compatible with classical surface accessibility calculations [66]. The Voronoi cells represent a convex polyhedron that contains all points of space closer to that atom than to any other atom. Two atoms are in contact if their Voronoi cells have a facet in common [66]. The overlap between the patch and the interface was calculated as the number of patch residues included in the interface divided by the total number of residues in the interface.

### Statistical Analysis

The *F*-test, Student's *t*-test (assuming equal variance), Mann–Whitney–Wilcoxson, and the Spearman correlation coefficient (CC) were performed using the R Stats package [67]. To account for multiple testing, the *P*-value was adjusted using the Bonferroni correction.

#### Support Vector Machine

SVM experiments were carried out with Gist Program version 2.1.1 (http://microarray.cpmc.columbia.edu/gist/). Input data were normalized by rescaling the columns to values between −1 and 1. A linear kernel was applied for all SVM classifiers. General tests were conducted by applying a “leave one out” cross-validation procedure. To evaluate the SVM performance, a ROC (receiver operating characteristic) curve describing the relationship between the false positive rate (FPR) and the true positive rate (TPR) was plotted. The area under the ROC curve (AUC) ranges between 0 to 1 and can be interpreted as the probability that when we randomly pick one positive and one negative example, the classifier will assign a higher score to the positive example than to the negative example. The AUCs are reported for each SVM test. In addition, we calculated the total accuracy, sensitivity, specificity and Matthews's correlation coefficient (MCC).
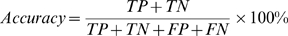


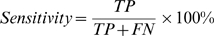


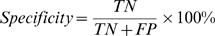


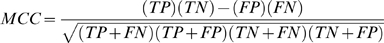



#### Feature selection

SVM-RFE feature selection method was applied for selecting the top ten features. RFE was originally proposed by Guyon et al. [68] to conduct gene selection for cancer classification. In the RFE algorithm, nested subsets of features are selected in a sequential backward elimination manner. At each step, the coefficients of the weight vector are used to compute the feature ranking score. In each of the iterations, 50% of the features with the lowest ranking scores were eliminated.

#### Multiclass SVM

The multiclass SVM approach, also called the one versus all approach [69], is generally a series of binary SVM classifiers, where in each classifier the members of one subclass (one) are separated from the rest of the data (all). Subsequently each member (protein) is held out from the training and tested against the different classifiers. The predicted subclass is defined according to the classifier for which the tested protein achieved the highest positive discriminating value. In the current study, we built three subclassifiers: (1) 46 rRNA-binding proteins against all other RBPs, (2) 23 mRNA-binding proteins against all other RBPs, (3) 13 tRNA-binding proteins against all other RBPs. For the multi-SVM experiment, we eliminated the viral RNA proteins that could not be classified into one of the three major groups. In addition, in order to have a reasonable number of RBPs in each subset, we extended the original set by adding new RBPs that do not share more than 25% sequence identity with the other proteins.

### Availability

A standalone package, NAbind, for nucleic-acid binding prediction (suitable for linux OS) is available for download (Dataset S2).

## Supporting Information

**Dataset S1** *P*-values are given for *F* and *t* tests (bold number denote statistically significant after Bonferroni correction).

**Dataset S2** RNbind Package-A standalone package for nucleic-acid-binding prediction (suitable for linux OS).

Figure S1 Spearman correlation coefficient values (ρ) calculated for each one of the 40 features comparing RBP vs. NNBP. The features are colored by group (detailed numbers are given in Dataset S1): Dark blue represents features related to the largest positive patch, in red are features related to the whole protein, in green are cleft-patch related features, and in cyan are the “other patches” features.
(0.90 MB TIF)

Table S1 Patch-interface overlap. Results are given for all protein-RNA complexes for which the interface could be defined. *Numbers denote number of residues.
(0.08 MB DOC)

Table S2 Patch interface overlap for positive and negative patches. Average and standard deviation of patch interface overlapping residues for ten positive patches and the largest negative patch. In the first row the number of overlapping residues is given. In the second and third rows are the normalized values, normalized to the interface and to the patch, respectively.
(0.03 MB DOC)

Table S3 RNA binding predictions for hypothetical proteins. The table summarizes the SVM results for the hypothetical RBPs that were verified experimentally to be involved in RNA-binding. Gene Ontology, protein function, structural motif, and SVM results are given. Shaded rows mark hypothetical RBPs that were predicted as non-RBPs.
(0.04 MB DOC)

Table S4 List of 76 representative RBPs grouped by family. *15 chains : 1fjgB 1fjgC 1fjgD 1fjgF 1fjgG 1fjgI 1fjgJ 1fjgL 1fjgM 1fjgN 1fjgO 1fjgP 1fjgR 1fjgS 1fjgT. ** 24 chains : 1jj21 1jj22 1jj2B 1jj2C 1jj2D 1jj2E 1jj2F 1jj2G 1jj2H 1jj2I 1jj2J 1jj2K 1jj2L 1jj2O 1jj2P 1jj2Q 1jj2R 1jj2T 1jj2U 1jj2V 1jj2W 1jj2X 1jj2Y 1jj2Z
(0.03 MB DOC)

Table S5 Detailed SVM results for “leave one out” vs. “leave family out” tests. *Numbers denote the discriminating value obtain from the SVM
(0.09 MB DOC)

Table S6 Detailed SVM results for the RRM family. Predictions are based on the discriminant value obtained by the SVM: 1 = predicted as an RBP ; −1 = predicted as NNBP; NA = could not be predicted based on SVM results.
(0.06 MB DOC)

Table S7 Mann-Whitney-Wilcoxon test results RRM-protein vs. RRM-RNA. Results of the Mann-Whitney-Wilcoxon test comparing the values of each of the 40 features between the RRM predicted as RBPs and the RRM predicted as NNBP.
(0.06 MB DOC)

Table S8 Multiclass SVM results. Multiclass SVM analysis for 3 subgroups: (A) mRNA, (B) rRNA, and (C) tRNA. Each protein in each of the subgroups was tested against the three different classifiers. The SVM results of each protein against the three different classifiers are given. A protein was classified into the subgroup in which it achieved the highest positive value, marked in red.
(0.16 MB DOC)
