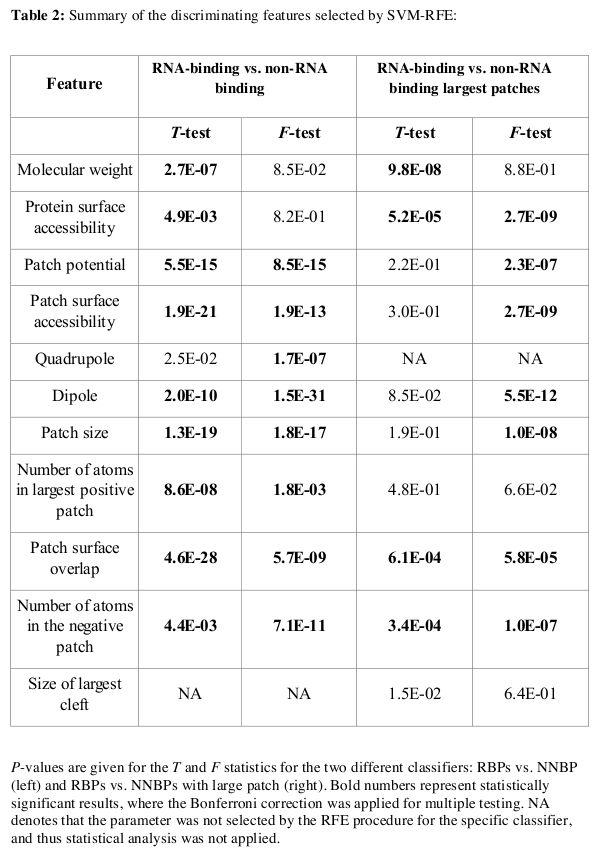# Correction: Classifying RNA-Binding Proteins Based on Electrostatic Properties

**DOI:** 10.1371/annotation/be4eb7dd-4092-49c1-a619-e8eeba40ed67

**Published:** 2008-08-21

**Authors:** Shula Shazman, Yael Mandel-Gutfreund

In Table 2, the headings F-test and T-test were swapped in the second and third columns. The second column should be T-test and the third should be F-test. Please see the corrected Table 2 here:

**Figure:**