# Supplementary figures and images for: Classifying RNA-Binding Proteins Based on Electrostatic Properties
Source: PLoS Comput Biol. 2008 Aug 8;4(8):e1000146. doi: 10.1371/journal.pcbi.1000146 (PMC2518515; doi:10.1371/journal.pcbi.1000146)

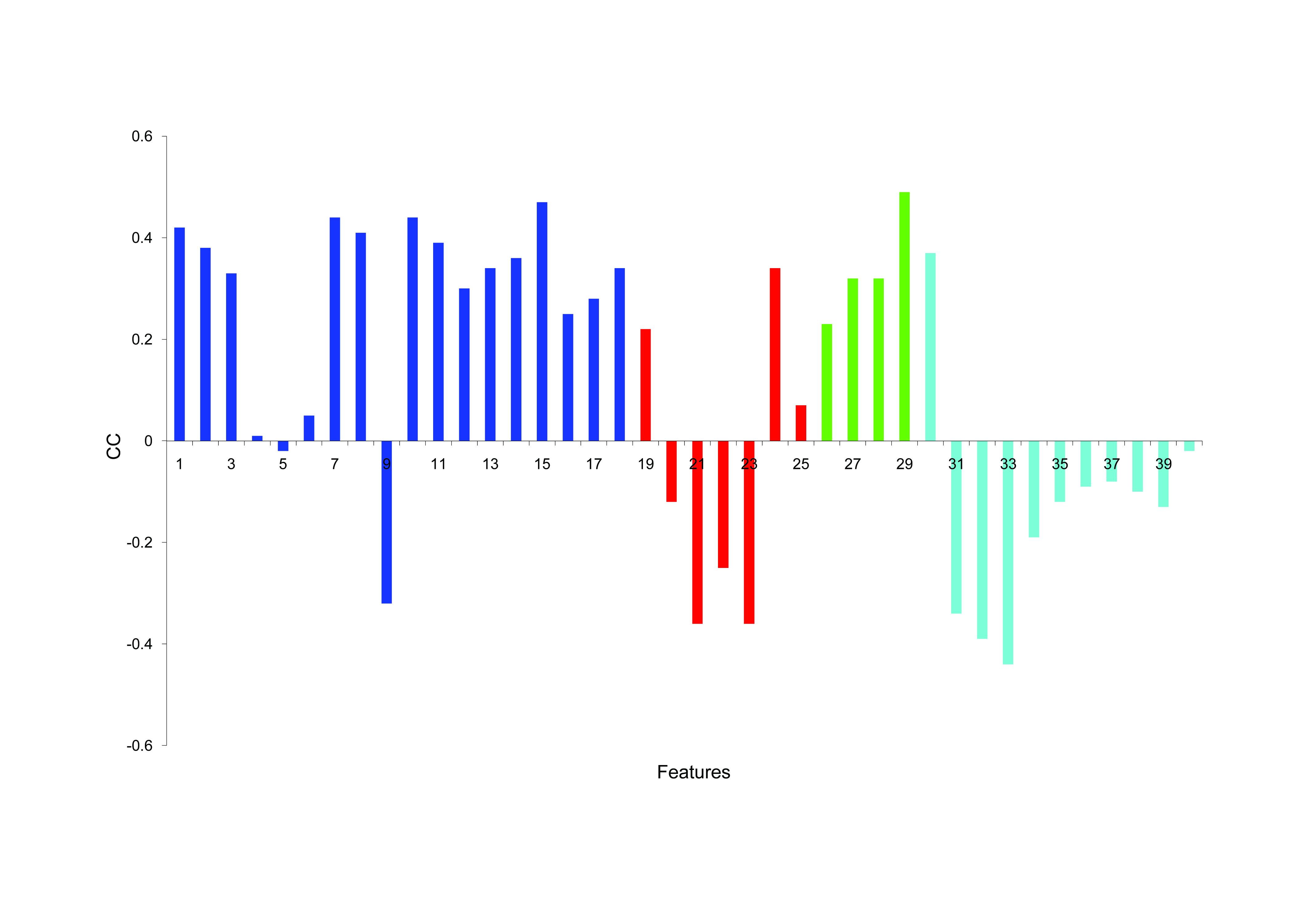

Supplement: Figure S1 — Spearman correlation coefficient values (ρ) calculated for each one of the 40 features comparing RBP vs. NNBP. The features are colored by group (detailed numbers are given in Dataset S1): Dark blue represents features related to the largest positive patch, in red are features related to the whole protein, in green are cleft-patch related features, and in cyan are the “other patches” features. (0.90 MB TIF) [file pcbi.1000146.s002.tif]
