# Supplementary material for: Classifying RNA-Binding Proteins Based on Electrostatic Properties
Source: PLoS Comput Biol. 2008 Aug 8;4(8):e1000146. doi: 10.1371/journal.pcbi.1000146 (PMC2518515; doi:10.1371/journal.pcbi.1000146)
Supplement: Table S1 — Patch-interface overlap. Results are given for all protein-RNA complexes for which the interface could be defined. *Numbers denote number of residues. (0.08 MB DOC) [file pcbi.1000146.s003.doc]

**Table S1: Patch-Interface overlap**

| **PDB code** | **Interface*** | **Patch*** | **Overlap*** | **Overlap/Interface** |
| --- | --- | --- | --- | --- |
| 1a1tA | 28 | 32 | 24 | 0.86 |
| 1a34A | 15 | 28 | 10 | 0.67 |
| 1a9nB | 37 | 48 | 29 | 0.78 |
| 1aq3A | 26 | 15 | 7 | 0.27 |
| 1b23P | 58 | 18 | 8 | 0.14 |
| 1ddlA | 22 | 9 | 0 | 0 |
| 1dfuP | 33 | 31 | 19 | 0.58 |
| 1e8oB | 9 | 32 | 2 | 0.22 |
| 1f7uA | 132 | 68 | 17 | 0.13 |
| 1fjgB | 30 | 49 | 19 | 0.63 |
| 1fjgC | 48 | 85 | 42 | 0.88 |
| 1fjgD | 82 | 129 | 75 | 0.92 |
| 1fjgF | 19 | 40 | 15 | 0.79 |
| 1fjgG | 19 | 90 | 1 | 0.05 |
| 1fjgI | 59 | 95 | 57 | 0.97 |
| 1fjgJ | 41 | 53 | 34 | 0.83 |
| 1fjgK | 44 | 52 | 28 | 0.64 |
| 1fjgL | 78 | 112 | 75 | 0.96 |
| 1fjgM | 58 | 90 | 58 | 1 |
| 1fjgN | 38 | 53 | 36 | 0.95 |
| 1fjgO | 46 | 64 | 37 | 0.80 |
| 1fjgP | 58 | 63 | 53 | 0.91 |
| 1fjgR | 22 | 50 | 20 | 0.91 |
| 1fjgS | 33 | 52 | 30 | 0.91 |
| 1fjgT | 60 | 75 | 55 | 0.92 |
| 1h2cA | 14 | 45 | 13 | 0.93 |
| 1hq1A | 26 | 24 | 15 | 0.58 |
| 1i6uA | 36 | 73 | 31 | 0.86 |
| 1jbrA | 35 | 9 | 8 | 0.23 |
| 1jidA | 38 | 66 | 36 | 0.95 |
| 1jj2B | 172 | 153 | 137 | 0.80 |
| 1jj2C | 131 | 110 | 98 | 0.75 |
| 1jj2D | 71 | 56 | 40 | 0.56 |
| 1jj2E | 46 | 6 | 2 | 0.04 |
| 1jj2F | 21 | 8 | 0 | 0 |
| 1jj2G | 13 | 16 | 11 | 0.85 |
| 1jj2H | 66 | 103 | 57 | 0.86 |
| 1jj2I | 71 | 66 | 54 | 0.76 |
| 1jj2J | 49 | 61 | 39 | 0.80 |
| 1jj2K | 84 | 29 | 29 | 0.35 |
| 1jj2L | 139 | 164 | 133 | 0.96 |
| 1jj2O | 96 | 62 | 54 | 0.56 |
| 1jj2P | 64 | 23 | 19 | 0.30 |
| 1jj2Q | 83 | 56 | 52 | 0.63 |
| 1jj2R | 34 | 27 | 22 | 0.65 |
| 1jj2T | 19 | 19 | 13 | 0.68 |
| 1jj2U | 27 | 35 | 25 | 0.93 |
| 1jj2V | 72 | 18 | 8 | 0.11 |
| 1jj2W | 40 | 23 | 16 | 0.4 |
| 1jj2Y | 38 | 68 | 37 | 0.97 |
| 1jj2Z | 54 | 49 | 48 | 0.89 |
| 1k8wA | 64 | 98 | 57 | 0.89 |
| 1knzA | 36 | 54 | 22 | 0.61 |
| 1kq2A | 7 | 14 | 2 | 0.29 |
| 1m8wA | 51 | 30 | 3 | 0.06 |
| 1mmsA | 36 | 101 | 35 | 0.97 |
| 1mzpA | 51 | 133 | 46 | 0.90 |
| 1q2rA | 54 | 69 | 33 | 0.61 |
| 1rgoA | 41 | 49 | 34 | 0.83 |
| 1si2A | 30 | 39 | 27 | 0.9 |
| 1un6B | 26 | 63 | 25 | 0.96 |
| 2fmtA | 62 | 90 | 45 | 0.73 |
| 2bggA | 58 | 205 | 57 | 0.98 |

Results are given for all protein-RNA complexes for which the interface could be defined. *Numbers denote number of residues
