# Supplementary material for: Classifying RNA-Binding Proteins Based on Electrostatic Properties
Source: PLoS Comput Biol. 2008 Aug 8;4(8):e1000146. doi: 10.1371/journal.pcbi.1000146 (PMC2518515; doi:10.1371/journal.pcbi.1000146)
Supplement: Table S2 — Patch interface overlap for positive and negative patches. Average and standard deviation of patch interface overlapping residues for ten positive patches and the largest negative patch. In the first row the number of overlapping residues is given. In the second and third rows are the normalized values, normalized to the interface and to the patch, respectively. (0.03 MB DOC) [file pcbi.1000146.s004.doc]

**Table S2: Patch interface overlap for positive and negative patches**

| N1 | P10 | P9 | P8 | P7 | P6 | P5 | P4 | P3 | P2 | P1 |  |
| --- | --- | --- | --- | --- | --- | --- | --- | --- | --- | --- | --- |
| 35.5±  39 | 1.9±  1.2 | 2±  1.3 | 2.3±  1.6 | 2.5±  1.7 | 2.8±  2.3 | 3.5±  2.8 | 5.2±  7.7 | 6.2±  8.4 | 10±  12.1 | 77±  45.4 | Patch Size |
| 0.12±  0.16 | 0.004±  0.009 | 0.009±  0.02 | 0.009±  0.03 | 0.01±  0.03 | 0.01±  0.03 | 0.02±  0.03 | 0.02±  0.08 | 0.03±  0.08 | 0.05±  0.01 | 0.68±  0.31 | Overlap/  Interface |
| 0.19±  0.24 | 0.17±  0.37 | 0.17±  0.36 | 0.19±  0.39 | 0.20±  0.39 | 0.21±  0.35 | 0.24±  0.36 | 0.21±  0.37 | 0.22±  0.38 | 0.19 ± 0.3 | 0.44 ± 0.24 | Overlap/  Patch |

Average and standard deviation of patch interface overlapping residues for ten positive patches and the largest negative patch. In the first row the number of overlapping residues is given. In the second and third rows are the normalized values, normalized to the interface and to the patch, respectively.
