# Supplementary material for: Classifying RNA-Binding Proteins Based on Electrostatic Properties
Source: PLoS Comput Biol. 2008 Aug 8;4(8):e1000146. doi: 10.1371/journal.pcbi.1000146 (PMC2518515; doi:10.1371/journal.pcbi.1000146)
Supplement: Table S3 — RNA binding predictions for hypothetical proteins. The table summarizes the SVM results for the hypothetical RBPs that were verified experimentally to be involved in RNA-binding. Gene Ontology, protein function, structural motif, and SVM results are given. Shaded rows mark hypothetical RBPs that were predicted as non-RBPs. (0.04 MB DOC) [file pcbi.1000146.s005.doc]

**Table S3: RNA binding predictions for hypothetical p**roteins

| **PDB code** | **Function** | **GO** | **SCOP** | **Prediction**  **Discriminant** |
| --- | --- | --- | --- | --- |
| 1dm9A | Recycling of free 50S ribosomal subunit | RNA-binding | Novel RNA-binding motif | 0.40 |
| 1gz0A | 23S rRNA methyltransferase | RNA-binding | RNA methyltransferase like | 0.32 |
| 1jkeA | D-tyrosyl-tRNA(Tyr) deacylase | Hydrolase activity | tRNA deacylase | -0.6 |
| 1mxiA | rRNA/tRNA methyltransferases | RNA-binding | rRNA/tRNA methyltransferases | 0.26 |
| 1nt2A | Involved in pre-rRNA and tRNA processing | RNA-binding | Fibrillarin homologue | -0.34 |
| 1nzjA | Glutamyl-Q tRNA(Asp) synthetase | Nucleotide binding | [aminoacyl-tRNA synthetases](http://www.rcsb.org/pdb/search/smartSubquery.do?smartSearchSubtype=TreeQuery&t=11&n=52375) | -0.35 |
| 1pc0A | Component of ribonuclease P complex | ribonuclease | RNase P | 0.24 |
| 1pxfA | tRNA binding protein | tRNA binding | OB fold | 0.22 |
| 1u04A | RISC complex | Nucleotide binding | PAZ domain | 0.59 |
| 1w9hA | Argonaute protein | Nucleic acid binding | PIWI domain | 0.33 |
| 1wi6A | Promotes exon skipping | NO | RRM | 0.54 |
| 1wy5A | **tRNA(Ile)-lysidine synthase** | NO | tRNA recognition domain | 0.16 |
| 2cqoA | Nucleolar protein | NO | S1 RNA binding domain | 0.88 |

The table summarizes the SVM results for the hypothetical RBPs that were verified experimentally to be involved in RNA-binding. Gene Ontology, protein function, structural motif and SVM results are given. Shaded rows mark hypothetical RBPs that were predicted as non-RBPs.
