# Supplementary material for: Classifying RNA-Binding Proteins Based on Electrostatic Properties
Source: PLoS Comput Biol. 2008 Aug 8;4(8):e1000146. doi: 10.1371/journal.pcbi.1000146 (PMC2518515; doi:10.1371/journal.pcbi.1000146)
Supplement: Table S4 — List of 76 representative RBPs grouped by family. *15 chains : 1fjgB 1fjgC 1fjgD 1fjgF 1fjgG 1fjgI 1fjgJ 1fjgL 1fjgM 1fjgN 1fjgO 1fjgP 1fjgR 1fjgS 1fjgT. ** 24 chains : 1jj21 1jj22 1jj2B 1jj2C 1jj2D 1jj2E 1jj2F 1jj2G 1jj2H 1jj2I 1jj2J 1jj2K 1jj2L 1jj2O 1jj2P 1jj2Q 1jj2R 1jj2T 1jj2U 1jj2V 1jj2W 1jj2X 1jj2Y 1jj2Z (0.03 MB DOC) [file pcbi.1000146.s006.doc]

**Table S4: List of 76** representative RBPs grouped by family

| Family name | Representative proteins |
| --- | --- |
| Zinc Finger motif | 1un6B 1rgoA 1altA |
| RRM motif | 1a9nB 1cx0_ |
| RNaze | 1k8wA 1a6f_ 1a2wA 1jbrA 1fjgK |
| PAZ domain | 1si2A 2bgg |
| Multimeric motifs | 1b34A 1m8wA 1gtfA 1kq2A |
| Capsid protein | 1aq3A 1a34A |
| SRP motif | 1e8oB 1hq1A 1jidA |
| Ribosomal | 1dfuP 1i6uA 1fjg* 1mzpA 1mmsA 1jj2** |
| tRNA binding proteins | 1asyA 1b23P 1f7uA 1q2rA 2fmtA |
| Other | 1ec6A 1ropA 1t4lB 1fukA 1knzA 1ddlA 1h2cA |

*15 chains : 1fjgB 1fjgC 1fjgD 1fjgF 1fjgG 1fjgI 1fjgJ 1fjgL 1fjgM 1fjgN 1fjgO 1fjgP 1fjgR 1fjgS 1fjgT.

** 24 chains : 1jj21 1jj22 1jj2B 1jj2C 1jj2D 1jj2E 1jj2F 1jj2G 1jj2H 1jj2I 1jj2J 1jj2K 1jj2L 1jj2O 1jj2P 1jj2Q 1jj2R 1jj2T 1jj2U 1jj2V 1jj2W 1jj2X 1jj2Y 1jj2Z
