# Supplementary material for: Classifying RNA-Binding Proteins Based on Electrostatic Properties
Source: PLoS Comput Biol. 2008 Aug 8;4(8):e1000146. doi: 10.1371/journal.pcbi.1000146 (PMC2518515; doi:10.1371/journal.pcbi.1000146)
Supplement: Table S5 — Detailed SVM results for “leave one out” vs. “leave family out” tests. *Numbers denote the discriminating value obtain from the SVM (0.09 MB DOC) [file pcbi.1000146.s007.doc]

**Table S5: Detailed SVM results for “leave one out” vs. “leave family out” tests**

| **PDB code** | **RNA ligand** | **Leave one out test*** | **Leave family out test*** |
| --- | --- | --- | --- |
| **Zinc Finger motif** |  |  |  |
| 1un6B | 5S rRNA | 1.54 | 1.51 |
| 1rgoA | mRNA | 1.26 | 1.22 |
| 1a1tA | Psi RNA | 1.57 | 1.53 |
|  |  |  |  |
| **RRM motif** |  |  |  |
| 1a9nB | U2snRNA | 0.65 | 0.64 |
| 1cx0_ | ribozyme | 0.67 | 0.65 |
|  |  |  |  |
| **RNaze** |  |  |  |
| 1k8wA | Stem-loop RNA of tRNA | -0.09 | -0.13 |
| 1a6f_ | tRNA precursor | 1.16 | 1.15 |
| 1a2wA | ssRNA /dsRNA | 0.32 | 0.29 |
| 1jbrA | SRL of 28S rRNA/tRNA | -0.57 | -0.59 |
| 1fjgK | rRNA | 0.53 | 0.5 |
|  |  |  |  |
| **PAZ domain** |  |  |  |
| 1si2A | siRNA | 0.24 | 0.28 |
| 2bggA | siRNA | -0.02 | 0.02 |
|  |  |  |  |
| **Multimeric motifs** |  |  |  |
| 1b34A | snRNA | 0.01 | 0.07 |
| 1kq2A | Viral mRNA | 0.02 | 0.08 |
| 1gtfA | Leader RNA | 0.28 | 0.35 |
| 1m8wA | 3’ UTR mRNA | -0.62 | -0.6 |
|  |  |  |  |
| **Capsid proteins** |  |  |  |
| 1aq3A | Viral RNA genome | -0.24 | -0.21 |
| 1a34A | Viral RNA | -0.05 | -0.02 |
|  |  |  |  |
| **SRP motif** |  |  |  |
| 1e8oB | 7S RNA | 0.93 | 0.91 |
| 1hq1A | 7S RNA | 0.22 | 0.18 |
| 1jidA | SRP RNA | 0.69 | 0.67 |
| **Ribosomal proteins** |  |  |  |
| 1iu6A | rRNA | 0.92 | 0.74 |
| 1fjgB | rRNA | -0.04 | -0.25 |
| 1fjgC | rRNA | 0.69 | 0.53 |
| 1fjgD | rRNA | 1.19 | 1.11 |
| 1fjgE | rRNA | 0.31 | 0.1 |
| 1fjgG | rRNA | 1.14 | 1.05 |
| 1fjgI | rRNA | 1.57 | 1.56 |
| 1fjgJ | rRNA | 1.28 | 1.21 |
| 1fjgL | rRNA | 1.76 | 1.71 |
| 1fjgM | rRNA | 1.89 | 1.99 |
| 1fjgN | rRNA | 1.81 | 1.71 |
| 1fjgO | rRNA | 1.13 | 0.96 |
| 1fjgP | rRNA | 1.23 | 1.11 |
| 1fjgR | rRNA | 1.26 | 1.12 |
| 1fjgS | rRNA | 1.41 | 1.29 |
| 1fjgT | rRNA | 1.79 | 1.82 |
| 1jj21 | rRNA | 2.02 | 1.96 |
| 1jj22 | rRNA | 1.19 | 1.06 |
| 1jj2B | rRNA | 1.07 | 1.04 |
| 1jj2C | rRNA | 0.92 | 0.84 |
| 1jj2D | rRNA | 0.96 | 0.83 |
| 1jj2E | rRNA | -0.00 | -0.22 |
| 1jj2F | rRNA | -0.28 | -0.53 |
| 1jj2G | rRNA | 1.39 | 1.28 |
| 1jj2H | rRNA | 1.04 | 0.91 |
| 1jj2I | rRNA | 0.92 | 0.82 |
| 1jj2J | rRNA | 0.71 | 0.52 |
| 1jj2K | rRNA | 1.01 | 0.99 |
| 1jj2L | rRNA | 1.66 | 1.65 |
| 1jj2O | rRNA | 1.48 | 1.53 |
| 1jj2P | rRNA | 0.42 | 0.23 |
| 1jj2Q | rRNA | 0.5 | 0.3 |
| 1jj2R | rRNA | 0.39 | 0.18 |
| 1jj2T | rRNA | 0.56 | 0.33 |
| 1jj2U | rRNA | 1.14 | 1.03 |
| 1jj2V | rRNA | -0.18 | -0.39 |
| 1jj2W | rRNA | 0.3 | 0.1 |
| 1jj2X | rRNA | 1.22 | 1.16 |
| 1jj2Y | rRNA | 1.64 | 1.53 |
| 1jj2Z | rRNA | 2 | 2.02 |
| 1dfuP | rRNA | 0.31 | 0.76 |
| 1mmsA | rRNA | 1.07 | 0.91 |
| 1mzpA | rRNA | 0.79 | 0.58 |
| **tRNA binding proteins** |  |  |  |
| 1asyA | tRNA | -0.17 | -0.09 |
| 1b23P | tRNA | -0.85 | -0.77 |
| 1f7uA | tRNA | -0.90 | -0.80 |
| 1q2rA | tRNA | -0.82 | -0.69 |
| 2fmtA | tRNA | -0.24 | -0.17 |
| **Others** |  |  |  |
| 1ec6A | mRNA | 0.96 | 0.96 |
| 1ropA | e.Coli plasmid RNA | 0.07 | 0.07 |
| 1t4lB | 5' terminal hairpin of snRNA precursor | 0.74 | 0.74 |
| 1fukA | 5'-UTR mRNA | -0.08 | -0.08 |
| 1knzA | rotavirus mRNA 3' | 0.61 | 0.61 |
| 1ddlA | Viral RNA | -0.26 | -0.26 |
| 1h2cA | E.coli RNA | 0.10 | 0.10 |

*****numbers denote the discriminating value obtain from the SVM
