# Supplementary material for: Classifying RNA-Binding Proteins Based on Electrostatic Properties
Source: PLoS Comput Biol. 2008 Aug 8;4(8):e1000146. doi: 10.1371/journal.pcbi.1000146 (PMC2518515; doi:10.1371/journal.pcbi.1000146)
Supplement: Table S6 — Detailed SVM results for the RRM family. Predictions are based on the discriminant value obtained by the SVM: 1 = predicted as an RBP ; −1 = predicted as NNBP; NA = could not be predicted based on SVM results. (0.06 MB DOC) [file pcbi.1000146.s001.doc]

**Table S6 : Detailed SVM results for the RRM family**

| Discriminant  value | SVM  Prediction | Structure  type | Protein description | PDB  code |
| --- | --- | --- | --- | --- |
| 0.21 | 1 | NMR | N-terminal RRM of Nucleolin rdb1 | 1fj7A |
| 0.15 | 1 | NMR | RRM of nucleolin in complex with RNA | 1fjcA |
| -0.39 | -1 | X-ray | An atypical RRM of Splicing Factor U2AF 35, involved in protein-protein interactions | 1jmtA |
| -0.16 | -1 | NMR | RRM of U2AF65 in complex with an N-terminal SF1 peptide | 1opiA |
| -0.22 | -1 | X-ray | The Y14 protein part of the Exon Junction Complex. RRM involved in protein interaction with Mago, RNA binding activity not confirmed | 1rk8A |
| 1.20 | 1 | NMR | Central RRM domain of human La protein involved in RNA binding | 1s79A |
| 0.90 | 1 | NMR | RNA binding domain from hypothetical protein BAB23448-poly A ribonuclease | 1whxA |
| 0.27 | 1 | NMR | The C-terminal RNA-binding domain of hnRNP D (AUF1) with telomere DNA | 1wtbA |
| 1.06 | 1 | X-ray | U1 snRNP RRM domain | 1zznA |
| 0.14 | 1 | NMR | RRM of squamous cell carcinoma antigen interacts with Tat RNA | 2do4A |
| 0.60 | 1 | NMR | RRM of tar DNA- binding protein-43 involved in CTFR splicing, binds specifically to UG repeated, | 2cqgA |
| 0.39 | 1 | NMR | RRM Domain in the Human Poly (ADP-ribose) Polymerase Family | 2dhxA |
| 0.76 | 1 | NMR | RRM in Insulin-like growth factor 2 mRNA binding protein 3 | 2e44A |
| 0.76 | 1 | NMR | RRM in **U6 snRNA-specific RNA uridyltransferase 1** | 2e5gA |
| 1.07 | 1 | NMR | RRM 2 in hnRNP L-like protein, suggested to bind RNA | 2e5iA |
| 0.98 | 1 | NMR | RRM of the REF2-I mRNA export factor | 2f3jA |
| 0.70 | 1 | NMR | RRM in the Human spliceosomal protein complex p14-SF3b155 | 2fhoB |
| 0 | NA | X-ray | RRM1 domain of U2AF65 variant bound to polypuridine tract | 2g4bA1 |
| 0.87 | 1 | X-ray | RRM2 domain of U2AF65 variant bound to polypuridine tract | 2g4bA2 |
| -0.29 | -1 | NMR | RRM and TAP binding domain of SRp20 protein bound to RNA | 2i2yA |
| -0.24 | -1 | X-ray | RRM of the SET1 Histone Methyltransferase | 2j8aA |
| 0.10 | 1 | NMR | RRM of elF3b and Its Interaction with eIF3j | 2nlwA |
| 1.33 | 1 | NMR | Structure of human SF2/ASF RNA RRM 2 | 2o3dA |
| -0.11 | -1 | X-ray | The U2AF-homology motif (UHM) of SPF45 mediates protein-protein interactions | 2pe8A |
| 0.40 | 1 | X-ray | RRM1 domain of the hnRNP A1 bound to ssDNA | 2up1A1 |
| 0.39 | 1 | X-ray | RRM2 domain of the hnRNP A1 bound to ssDNA | 2up1A2 |
| 0.06 | 1 | NMR | RRM of the pre-mRNA- splicing factor rbm22 | 2ytcA |

Predictions are based on the discriminant value obtain by the SVM: 1 = predicted as an RBP ; -1= predicted as NNBP; NA = could not be predicted based on SVM results.
