# Supplementary material for: Classifying RNA-Binding Proteins Based on Electrostatic Properties
Source: PLoS Comput Biol. 2008 Aug 8;4(8):e1000146. doi: 10.1371/journal.pcbi.1000146 (PMC2518515; doi:10.1371/journal.pcbi.1000146)
Supplement: Table S7 — Mann-Whitney-Wilcoxon test results RRM-protein vs. RRM-RNA. Results of the Mann-Whitney-Wilcoxon test comparing the values of each of the 40 features between the RRM predicted as RBPs and the RRM predicted as NNBP. (0.06 MB DOC) [file pcbi.1000146.s008.doc]

**Table S7: Mann-Whiteny-Wilcoxon test results RRM-protein vs. RRM-RNA**

| **Parameter** | **p-value** |
| --- | --- |
| **I. Largest patch parameters** |  |
| **1.** Patch size | 0.005 |
| **2.** Patch potential | 0.024 |
| **3.** Number of residues in patch | 0.012 |
| **4.** Beta in patch | 0.292 |
| **5.** Helix in patch | 0.683 |
| **6.** Loop in patch | 0.621 |
| **7.** Patch surface accessibility | 0.0007 |
| **8.** Surface accessibility per residue | 0.029 |
| **9.** Patch roughness | 0.621 |
| **10.** Number of lys in patch | 0.614 |
| **11.** Number of arg in patch | 0.095 |
| **12.** Number of polar amino acid in patch | 0.681 |
| **13.** Atoms in patch1 (largest patch) | 0.008 |
| **14.** Hydrogen bond potential acceptors | 0.002 |
| **15.** Hydrogen bond potential donors | 0.002 |
| **16.** Satisfied Acceptors | 0.092 |
| **17.** Satisfied Donors | 0.362 |
| **18.** Hydrogen bond in patch | 0.496 |
|  |  |
| **II. Protein parameters** |  |
| **19.** Molecular weights per residue | 0.192 |
| **20.** Radius of gyration | 0.272 |
| **21.** Normal gyration radius | 0.003 |
| **22.** Protein surface accessibility | 0.408 |
| **23.** Molecular weights | 0.192 |
| **24.** Dipole moment | 0.610 |
| **25.** Quadrapole moment | 0.027 |
|  |  |
| **III. Cleft-Patch parameters** |  |
| **26.** Largest cleft-patch overlap | 0.095 |
| **27.** Second cleft-patch overlap | 0.013 |
| **28.** Third cleft-patch overlap | 0.810 |
| **29.** Total clefts – patch overlap | 0.024 |
|  |  |
| **IV. Other patches** |  |
| **30.** Number of residues in lysout patch | 0.865 |
| **31.** Number of residues in negative patch | 0.518 |
| **32.** Atoms in patch2 (second largest patch) | 0.892 |
| **33.** Atoms in patch3 (third largest patch) | 1 |
| **34.** Atoms in negative patch | 0.174 |
| **35.** Distance patch1 patch2 | 0.973 |
| **36.** Distance patch1 patch3 | 0.812 |
| **37.** Distance patch2 patch3 | 0.144 |
| **38.** Distance patch1 negative patch | 0.973 |
| **39.** Distance patch2 negative patch | 0.919 |
| **40.** Distance patch3 negative patch | 0.396 |

Results of the Mann-Whitney-Wilcoxon test comparing the values of each of the 40 features between the RRM predicted as RBPs and the RRM predicted as NNBP.
