# Supplementary material for: Classifying RNA-Binding Proteins Based on Electrostatic Properties
Source: PLoS Comput Biol. 2008 Aug 8;4(8):e1000146. doi: 10.1371/journal.pcbi.1000146 (PMC2518515; doi:10.1371/journal.pcbi.1000146)
Supplement: Table S8 — Multiclass SVM results. Multiclass SVM analysis for 3 subgroups: (A) mRNA, (B) rRNA, and (C) tRNA. Each protein in each of the subgroups was tested against the three different classifiers. The SVM results of each protein against the three different classifiers are given. A protein was classified into the subgroup in which it achieved the highest positive value, marked in red. (0.16 MB DOC) [file pcbi.1000146.s009.doc]

**Table S8 – Multi-class SVM results**

| *PDB*  *Code* | *Function* | *RNA-binding motif (based on Pfam)* | | | *Structural motif*  *(based on SCOP)* | *mRNA vs all* | *rRNA vs all* | *tRNA vs all* |
| --- | --- | --- | --- | --- | --- | --- | --- | --- |
| **tRNA** | | | | | | | | |
| 1asyA | Class II aminoacyl transfer RNA synthetases | | tRNA_anticodon binding domain  tRNA synthetases class II | OB-fold | | -0.16 | -0.21 | 0.25 |
| 1b23P | Elongation factor | | Elongation factor GTP binding domain | -P-loop containing nucleoside triphosphate hydrolases | | -0.07 | -0.58 | 0.83 |
| 1f7uA | Arginyl-tRNA synthetase, cytoplasmic | | tRNA synthetases class I | Anticodon binding domain | | 0.07 | -0.7 | 0.74 |
| 1k8wA | tRNA pseudouridine synthase B | | TruB family pseudouridylate synthase | [PUA domain-like](http://www.rcsb.org/pdb/search/smartSubquery.do?smartSearchSubtype=TreeQuery&t=11&n=88696) | | -0.19 | -0.13 | 0.12 |
| 1q2rA | Queuine tRNA-ribosyltransferase | | TGT | TIM beta/alpha-barrel | | -0.14 | -0.5 | 0.73 |
| 2fmtA | methionyl-tRNAfMet transformylase | |  | -[FMT C-terminal domain-like](http://www.rcsb.org/pdb/search/smartSubquery.do?smartSearchSubtype=TreeQuery&t=11&n=50485)  -Formyltransferase | | -0.02 | -0.34 | 0.17 |
| 1c0aA | Aspartyl-tRNA synthetase | | tRNA_anticodon binding domain  tRNA synthetases class II | Beta Barrel | | 0.03 | -0.51 | 0.47 |
| 1h3eA | Tyrosyl-tRNA synthetase | | tRNA synthetases class I | [RN](http://www.rcsb.org/pdb/search/smartSubquery.do?smartSearchSubtype=TreeQuery&t=11&n=55173)A binding domain | | -0.11 | -0.40 | 0.40 |
| 1j2bA | 7-cyano-7-deazaguanine tRNA-ribosyltransferase | | TGT  PUA domain | -[PUA domain-like](http://www.rcsb.org/pdb/search/smartSubquery.do?smartSearchSubtype=TreeQuery&t=11&n=88696)  -TIM barrel  -Cystatin-like | | -0.40 | -0.04 | 0.30 |
| 1n78A | Glutamyl-tRNA synthetase | | tRNA synthetases  class I | -Anticodon-binding domain  -Nucleotide alpha hydrolase-like | | -0.01 | -0.57 | 0.65 |
| 1qf6A | Threonyl-tRNA synthetase | | tRNA synthetases class II | -Anticodon binding domain**-**like  -Beta-Grasp (ubiquitin-like) | | -0.14 | -0.38 | 0.51 |
| 1qtqA | Glutaminyl-tRNA synthetase | | tRNA synthetases  class I | -[Ribosomal protein L25-like](http://www.rcsb.org/pdb/search/smartSubquery.do?smartSearchSubtype=TreeQuery&t=11&n=50714)  -hydrolase-like | | -0.38 | -0.23 | 0.46 |
| 1serA | Seryl-tRNA synthetase | | tRNA synthetases  class I | -Long alpha-hairpin  -biotin synthetases | | 0.20 | -0.87 | 0.83 |
| **rRNA** | | | | | | | | |
| 1a6f_ | Ribonuclease P protein component | Ribonuclease P | | | [Ribosomal protein S5 -like](http://www.rcsb.org/pdb/search/smartSubquery.do?smartSearchSubtype=TreeQuery&t=11&n=54210) | -0.77 | 0.75 | -0.78 |
| 1dfuP | 50S ribosomal protein L25 | Ribosomal L25p | | | [Ribosomal protein L25-like](http://www.rcsb.org/pdb/search/smartSubquery.do?smartSearchSubtype=TreeQuery&t=11&n=50714) | 0.20 | -0.27 | -0.16 |
| 1fjgB | 30s ribosomal protein | Ribosomal protein S2 | | | Flavodoxin-like | -0.03 | -0.42 | 0.32 |
| 1fjgC | 30s ribosomal protein | Ribosomal protein S3  KH domain | | | Protease prodomain-like. | -0.30 | 0.13 | -0.29 |
| 1fjgD | 30s ribosomal protein | Ribosomal protein S4 | | | Alpha-L RNA-binding motif | -0.74 | 0.69 | -0.66 |
| 1fjgF | 30s ribosomal protein | Ribosomal protein S6  Coiled Coil | | | Ferredoxin-like | 0.06 | -0.30 | -0.06 |
| 1fjgG | 30s ribosomal protein | Ribosomal protein S7 | | | Ribosomal protein –S7 like | -0.36 | 0.47 | -0.72 |
| 1fjgI | 30s ribosomal protein | Ribosomal protein S9 | | | OB-fold | -0.75 | 0.86 | -1.01 |
| 1fjgJ | 30s ribosomal protein | Ribosomal protein S10 | | | Ferredoxin-like | -0.27 | 0.43 | -0.89 |
| 1fjgK | 30s ribosomal protein | Ribosomal protein S11 | | | Ribonuclease H-like motif | 0.15 | -0.11 | -0.50 |
| 1fjgL | 30s ribosomal protein | Ribosomal protein S12 | | | OB-fold | -0.69 | 1.00 | -1.33 |
| 1fjgM | 30s ribosomal protein | Ribosomal protein S13 | | | H2TH domain | -1.01 | 1.26 | -1.26 |
| 1fjgN | 30s ribosomal protein | Ribosomal protein S14 | | | Glucocorticoid receptor like (DNA Binding domain) | -0.34 | 0.89 | -1.58 |
| 1fjgO | 30s ribosomal protein | Ribosomal protein S15 | | | RNA-binding domain | -0.04 | 0.30 | -0.87 |
| 1fjgP | 30s ribosomal protein | Ribosomal protein S16 | | | Ribosomal protein –S16 like | -0.55 | 0.63 | -0.90 |
| 1fjgR | 30s ribosomal protein | Ribosomal protein S18 | | | DNA/RNA binding | -0.31 | 0.49 | -1.01 |
| 1fjgS | 30s ribosomal protein | Ribosomal protein S19 | | | Ribosomal protein S19**-like** | -0.23 | 0.50 | -1.18 |
| 1fjgT | 30s ribosomal protein | Ribosomal protein S20p | | | Spectrin repeat-like | -0.91 | 1.16 | -1.32 |
| 1i6uA | ribosomal protein s8/rrna complex | Ribosomal S8 | | | Ribosomal protein-S8 like | -0.29 | 0.30 | -0.54 |
| 1jbrA | ribotoxin restrictocin and RNA inhibitor | Signal Peptide  Ribonuclease | | | Microbial Ribonucleases | 0.24 | -0.76 | 0.47 |
| 1jj21 | large ribosomal subunit | -- | | | Non-globular | -0.24 | 0.91 | -1.77 |
| 1jj22 | large ribosomal subunit | Ribosomal L44 | | | Rubredoxin-like | 0.06 | 0.21 | -0.95 |
| 1jj2B | large ribosomal subunit | -- | | | elongation factor common domain | -0.76 | 0.58 | -0.50 |
| 1jj2C | large ribosomal subunit | -- | | | Ribosomal protein-L4 like | -0.29 | 0.29 | -0.48 |
| 1jj2D | Large ribosomal subunit | -- | | | Ribosomal protein-L5 like | -0.15 | 0.22 | -0.64 |
| 1jj2E | large ribosomal subunit | -- | | | Ribosomal protein-L6 like | 0.05 | -0.51 | 0.34 |
| 1jj2F | large ribosomal subunit | Ribosomal L7Ae | | | chorismate mutase-like | 0.18 | -0.65 | 0.47 |
| 1jj2G | large ribosomal subunit | -- | | | Ribosomal protein- L10 like | 0.31 | 0.25 | -1.28 |
| 1jj2H | large ribosomal subunit | -- | | | Hammerhead | -0.63 | 0.60 | -0.61 |
| 1jj2I | large ribosomal subunit | -- | | | Ribosomal protein-L13 like | -0.33 | 0.29 | -0.49 |
| 1jj2J | large ribosomal subunit |  | | | Ribosomal protein-L14 like | -0.22 | 0.11 | -0.36 |
| 1jj2K | large ribosomal subunit | L15 | | | Ribosomal proteins –L15 like | 0.03 | 0.12 | -0.73 |
| 1jj2L | large ribosomal subunit | KOW | | | Ribosomal proteins | -0.97 | 1.15 | -1.10 |
| 1jj2O | large ribosomal subunit | -- | | | Ribosomal protein- L19 like | -0.60 | 0.67 | -0.88 |
| 1jj2P | large ribosomal subunit | -- | | | SH3-like barrel | 0.33 | -0.34 | -0.29 |
| 1jj2R | large ribosomal subunit | -- | | | Ribosomal proteins S24e, L23 and L15e | 0.21 | -0.33 | -0.20 |
| 1jj2T | large ribosomal subunit | [Ribosomal L24e](http://www.sanger.ac.uk/cgi-bin/Pfam/getacc?PF01246) | | | Glucocorticoid receptor-like (DNA Binding Domain) | 0.38 | -0.34 | -0.49 |
| 1jj2U | large ribosomal subunit | -- | | | Long alpha-hairpin | -0.13 | 0.29 | -0.86 |
| 1jj2V | large ribosomal subunit | -- | | | Ribosomal protein | 0.10 | -0.59 | 0.45 |
| 1jj2W | large ribosomal subunit | -- | | | Ribosomal protein | 0.21 | -0.35 | -0.11 |
| 1jj2X | large ribosomal subunit | -- | | | Barstar-like | -0.22 | 0.44 | -0.85 |
| 1jj2Y | large ribosomal subunit | -- | | | Rubredoxin-like | -0.48 | 0.84 | -1.37 |
| 1jj2Z | large ribosomal subunit | -- | | | Rubredoxin-like | -0.68 | 1.17 | -1.56 |
| 1mmsA | ribosomal protein l11-RNA complex | [Ribosomal L11](http://www.sanger.ac.uk/cgi-bin/Pfam/getacc?PF00298) | | | DNA/RNA-binding Ribosomal protein | -0.36 | 0.34 | -0.70 |
| 1mzpA | l1 protuberance in the ribosome | [Ribosomal L1](http://www.sanger.ac.uk/cgi-bin/Pfam/getacc?PF00687) | | | Ribosomal-L1like | -0.38 | 0.25 | -0.46 |
| 1un6B | Transcription factor iiia | zinc finger | | | [C2H2 and C2HC zinc fingers](http://www.rcsb.org/pdb/search/smartSubquery.do?smartSearchSubtype=TreeQuery&t=11&n=57666) | -0.43 | 0.64 | -1.14 |
| **mRNA** | | | | | | | | |
| 2a8vA | Rho transcription termination factor | Rho_N | | | OB-fold | 0.29 | -0.66 | 0.23 |
| 1fxlA | C-fos RNA  binds AU rich sequences | RRM_1 | | | Ferredoxin-like | -0.12 | 0.05 | -0.40 |
| 1a9nB | U2 RNA hairpin | LRR_1 | | | Ferredoxin-like | -0.34 | 0.31 | -0.53 |
| 1b34A | snRNP sm d1 | LSM | | | Sm-like fold | 0.43 | -0.49 | -0.26 |
| 1ec6A | NOVA-2 | KH_1 | | | KH-domain  (KH type I) | -0.16 | 0.31 | -0.80 |
| 1gtfA | trp RNA-binding attenuation | Trp_BP | | | Double-stranded beta-helix | 0.38 | -0.32 | -0.46 |
| 1knzA | Binds rotavirus mRNA 3' | Rota_NSP3  Coiled coil | | | NSP3 homodimer | 0.25 | -0.08 | -0.61 |
| 1kq2A | HFQ-RNA complex | LSM | | | Sm-like fold | 0.31 | -0.39 | -0.21 |
| 1m8wA | Human pumilio1 | PUF | | | Alpha-alpha superhelix | -0.08 | -0.59 | 0.64 |
| 1rgoA | Butyrate response factor 2 | zf-CCCH  [Tis11B_N](http://www.sanger.ac.uk/cgi-bin/Pfam/getacc?PF04553) | | | CCCH zinc finger | 0.03 | 0.40 | -1.21 |
| 1fj7A | Nucleolin rbd1 | RRM_1 | | | Ferredoxin-like | 0.41 | -0.35 | -0.39 |
| 1fjcA | Nucleolin rbd2 | RRM_1 | | | Ferredoxin-like | 0.44 | -0.42 | -0.24 |
| 1fo1A | Nuclear RNA export factor 1 | [Tap-RNA_bind](http://www.sanger.ac.uk/cgi-bin/Pfam/getacc?PF09162)  LRR_1 | | | LRR  Ferredoxin-like | 0.09 | -0.30 | 0.05 |
| 1jmtB | Splicing factor u2af 35 | [zf-CCCH](http://www.sanger.ac.uk/cgi-bin/Pfam/getacc?PF00642)  [RRM_1](http://www.sanger.ac.uk/cgi-bin/Pfam/getacc?PF00076) | | | **--** | 0.67 | -0.04 | -1.23 |
| 1owxA | Lupus la protein | La  RRM_1 | | | Ferredoxin-like | 0.52 | -0.46 | -0.24 |
| 1s7aA | Lupus la protein | La motif | | | [DNA/RNA-binding](http://www.rcsb.org/pdb/search/smartSubquery.do?smartSearchSubtype=TreeQuery&t=11&n=46688) | 0.04 | -0.09 | -0.39 |
| 1whvA | Hydrolase | RNA_bind | | | Ferredoxin-like | 0.49 | -0.36 | -0.55 |
| 1whyA | Exonucleolytic degradation of the poly(A) tail | RRM_1 | | | Ferredoxin-like | 0.24 | -0.01 | -0.82 |
| 1wi6A | Protein raver-1 | RRM_1 | | | Ferredoxin-like | 0.47 | -0.24 | -0.65 |
| 1wwhA | Nucleoporin 35 | [MPPN](http://www.sanger.ac.uk/cgi-bin/Pfam/getacc?PF05172) | | | Ferredoxin-like | 0.01 | -0.07 | -0.38 |
| 2ytcA | Pre-mRNA-splicing factor rbm22 | [--](http://www.sanger.ac.uk/cgi-bin/Pfam/getacc?PF05172) | | | **--** | 0.39 | -0.44 | -0.11 |
| 2qndA | Human fragile X mental retardation protein | KH-1  KH-2 | | | **--** | 0.39 | -0.67 | 0.26 |
| 2py9A | Protein-RNA interaction, poly(c)-binding protein-2 | KH-1 | | | Eukaryotic type KH-domain (KH- type I) | 0.02 | 0.21 | -0.85 |

Multiclass SVM analysis for 3 subgroups: A. mRNA B. rRNA and C. tRNA.

Each protein in each of the subgroups was tested against the three different classifiers. The SVM results of each protein against the three different classifiers are given. A protein was classified into the subgroup in which it achieved the highest positive value, marked in red.
